# Lysine‐specific demethylase 1 deletion reshapes tumour microenvironment to overcome acquired resistance to anti‐programmed death 1 therapy in liver cancer

**DOI:** 10.1002/ctm2.70335

**Published:** 2025-05-12

**Authors:** Chen Liang, Mu Ye, Lei Yu, Peng‐Fei Zhang, Xiao‐Jun Guo, Xian‐Long Meng, Hai‐Ying Zeng, Shu‐Yang Hu, Dao‐Han Zhang, Qi‐Man Sun, Ying‐Hao Shen, Jia‐Bin Cai, Shuang‐Qi Li, Zhen Chen, Ying‐Hong Shi, Ai‐Wu Ke, Yujiang G. Shi, Jian Zhou, Jia Fan, Fei‐Zhen Wu, Xiao‐Yong Huang, Guo‐Ming Shi, Zheng Tang, Jia‐Cheng Lu

**Affiliations:** ^1^ Department of Liver Surgery and Transplantation Zhongshan Hospital, Fudan University Shanghai China; ^2^ Shanghai Institute of Infectious Disease and Biosecurity Fudan University Shanghai China; ^3^ Liver Cancer Institute Zhongshan Hospital, Fudan University Shanghai China; ^4^ Key Laboratory of Carcinogenesis and Cancer Invasion, Ministry of Education of the People's Republic of China Shanghai China; ^5^ Department of Medical Oncology Shanghai Geriatric Medical Center (Zhongshan Hospital, Fudan University Minhang Meilong) Shanghai China; ^6^ Department of Pathology Zhongshan Hospital, Fudan University Shanghai China; ^7^ Key Laboratory of Medical Epigenetics and Metabolism Institutes of Biomedical Sciences Fudan University Shanghai China; ^8^ Clinical Research Unit, Institute of Clinical Science Zhongshan Hospital, Fudan University Shanghai China

**Keywords:** acquired resistance, hepatocellular carcinoma, immune checkpoint blockade, lysine‐specific demethylase 1, programmed death ligand 1, programmed death protein 1, tumour microenvironment

## Abstract

**Background:**

Immune checkpoint blockade, particularly targeting programmed death 1 (PD‐1) and programmed death ligand 1 (PD‐L1), shows promise in treating hepatocellular carcinoma (HCC). However, acquired resistance, especially in patients with ‘hot tumours’, limits sustained benefits. Lysine‐specific demethylase 1 (LSD1) plays a role in converting ‘cold tumours’ to ‘hot tumours’, but its involvement in PD‐1 inhibitor resistance in HCC is unclear.

**Methods:**

LSD1 and PD‐L1 expression, along with CD8^+^ T cell infiltration, were assessed using immunohistochemistry in HCC tissues, correlating these markers with patient prognosis. The impact of LSD1 deletion on tumour cell proliferation and CD8^+^ T cell interactions was examined in vitro. Mouse models were used to study the combined effects of LSD1 inhibition and anti‐PD‐1 therapy on tumour growth and the tumour microenvironment (TME). The clinical relevance of LSD1, CD74 and effector CD8^+^ T cells was validated in advanced HCC patients treated with PD‐1 blockade.

**Results:**

LSD1 overexpression in HCC patients correlated with reduced PD‐L1 expression, less CD8^+^ T cell infiltration and poorer prognosis. LSD1 deletion increased PD‐L1 expression, boosted effector CD8^+^ T cells in vitro and inhibited tumour growth in vivo. While anti‐PD‐1 monotherapy initially suppressed tumour growth, it led to relapse upon antibody withdrawal. In contrast, combining LSD1 inhibition with anti‐PD‐1 therapy effectively halted tumour growth and prevented relapse, likely through TME remodelling, enhanced CD8^+^ T cell activity and improved CD74‐mediated antigen presentation. Clinically, low LSD1 expression was associated with better response to anti‐PD‐1 therapy.

**Conclusion:**

LSD1 deletion reshapes the TME, enhances CD8^+^ T cell function and prevents acquired resistance to anti‐PD‐1 therapy in HCC. The combination of LSD1 inhibitors and PD‐1 blockade offers a promising strategy for overcoming resistance in advanced HCC.

**Key points:**

- Uncovering the synthetic lethality resulting from LSD1 deletion and PD1 inhibitor co‐administration, evaluating their combined effects on tumour growth and TME remodelling.
- Elucidating the mechanism underlying the combined therapy of LSD1 deletion with PD1 inhibition for HCC.
- Exploring the implications of LSD1, CD74 and effector CD8^+^ T cell expression levels in advanced HCC patients undergoing anti‐PD1 treatment.

## Graphical abstract

LSD1 deletion increased PD‐L1 expression in tumor cells, rejuvenated effector CD8+ T cells, and enhanced antigen presentation. Combination therapy with an LSD1 inhibitor and anti‐PD1 antibody remodeled the tumor microenvironment, reversing the acquired resistance and prolonging the duration of response to anti‐PD1 therapy.

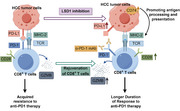

## 1 INTRODUCTION

Hepatocellular carcinoma (HCC) stands as one of the main causes of cancer‐related mortality on a global scale.^1^ Owing to the high recurrent rate after operation and early metastasis at the time of diagnosis, only a small fraction of patients with HCC benefit from surgical intervene.^2^ Notably, despite the approval of standard systemic treatments for advanced HCC, including multi‐kinase inhibitors such as Sorafenib, Lenvatinib and Regorafenib, these medications confer only modest survival advantages for individuals with advanced HCC.^3^ This challenging scenario underscores the imperative to explore novel and more efficacious treatments for HCC.

Programmed death 1 (PD1), recognized as a pivotal immune checkpoint molecule, engages with programmed death ligands L1/L2 (PD‐L1/L2) to transmit an inhibitory signal, thereby suppressing effector function and allowing cancer cells to evade tumour immunity.^4^ Monoclonal antibodies targeting the immune checkpoint molecule PD1 can interrupt the PD1/PD‐L1/PD‐L2 signal on effector immune cells, such as CD8^+^ T cells, leading to anti‐tumour activity.^5^ Consequently, these antibodies have gained approval for treating various malignancies, including HCC.^6^, ^7^, ^8^, ^9^ However, the efficacy of PD1/PD‐L1 antibodies is limited, as only a minority of patients experience benefits due to a low tumour response rate and the emergence of acquired resistance.^10^ Recent attention has been directly paid towards the role of epigenetic modifiers in immunotherapy. Pioneering research has shown that lysine‐specific demethylase 1 (LSD1, also known as KDM1A) suppressed endogenous double‐stranded RNA (dsRNA) and interferon (IFN) responses in tumour cells.^11^ Depletion of LSD1 renders non‐responsive refractory B16 tumours significantly sensitive to anti‐PD1 therapy. Another study reported that a pretreated combined therapy of a DNA methyltransferase inhibitor with intermittent histone deacetylase inhibitor substantially extends the duration of response (DoR) to immune checkpoint blockade (ICB) in advanced non‐small‐cell lung cancer patients.^12^ Therefore, identifying rational combinations to enhance anti‐tumour immunity and improve the response to ICB remains a prominent challenge in the field.

LSD1 stands as the pioneering histone demethylase first identified for its capacity to regulate gene expression in various contexts, including embryonic stem cells, hematopoietic stem cells and cancers.^13^ Functionally, LSD1 plays a transcriptionally repressive role through the demethylation of active histones H3K4me1 and H3K4me2, while acting as a gene activator by demethylating histones H3K9me1 and H3K9me2. Overexpression of LSD1 is observed in numerous human cancers, including HCC,^14^ and has been positively correlated with poor prognosis in patients with malignant tumours.^15^, ^16^ Experimental strategies, such as the knockdown of LSD1 expression using small‐interfering RNAs, demonstrate inhibitory effects on oncogenes, reducing the growth rate, migration and invasion abilities of cancer cells while restoring therapeutic sensitivity. Moreover, LSD1 inhibitors exhibit the capacity to reactivate silenced tumour suppressors in cancer.^17^, ^18^, ^19^ Notably, LSD1 also assumes a repressive role in the expression of pro‐inflammatory cytokines, including IL1α, IL1β, IL6 and IL8, suggesting that LSD1 inhibition may favour the restoration of anti‐tumour immunity.^20^ Collectively, these findings underscore the significant role of the histone demethylase LSD1 in the intrinsic resistance to immune checkpoint inhibitors (ICIs). However, its exact role and molecular mechanisms in acquired resistance to ICIs, particularly in the context of treating HCC, warrant further comprehensive investigation.

In the present study, our investigation began by exploring the correlation between the expression levels of LSD1, PD‐L1, the number of CD8^+^ T cells, along with their clinical significance. Subsequently, we conducted an examination of the impact of LSD1 inhibition on tumour cell growth, the quantity and functionality of CD8^+^ T cells and their interactive dynamics in vitro. Employing animal models that mimic diverse tumour microenvironment (TME), we further scrutinized the synthetic lethality resulting from LSD1 deletion and PD1 inhibitor co‐administration, evaluating their combined effects on tumour growth and the remodelling of the TME in vivo. The molecular mechanism underlying the combined therapy of LSD1 deletion with anti‐PD1 antibody for liver cancer was also elucidated in this study. Finally, we delved into the implications of the expression levels of LSD1, CD74 and effector CD8^+^ T cells in advanced HCC patients who underwent PD1 inhibitor treatment.

## 2 MATERIALS AND METHODS

### 2.1 Tumour samples collection and patient's follow‐up

Fresh tumour samples taken from areas adjacent to the margin of tumours were obtained from 206 consecutive patients with HCC who underwent curative resection between 2005 and 2008 at the Liver Cancer Institute of Fudan University (Shanghai, China). Paraffin blocks were selected only based on the availability of suitable formalin‐fixed, paraffin‐embedded tissue, complete clinicopathologic and follow‐up data for the patients. Ethical approval was obtained from the Zhongshan Hospital Research Ethics Committee (Y2019‐194), and written informed consent was obtained from each patient.

### 2.2 Tissue microarray and immunohistochemistry

A tissue microarray (TMA) was constructed by Shanghai Biochip Co. Ltd. Immunohistochemistry (IHC) was performed according to the manufacturer's instruction as described previously.^21^ Images were captured with Leica Q Win Plus v3 software (Leica Microsystems Imaging Solutions). For multiple immunofluorescence, antigen was retrieved at ethylenediaminetetraacetic acid (EDTA) antigen retrieval buffer (pH 8.0) and maintained at a boiling temperature for 6 min, and then another sub‐boiling temperature for 8 min. After fluorescence quenching, the samples were blocked in 5% normal goat serum in phosphate‐buffered saline (PBS) with .25% Triton X‐100 (PBST) for 30 min at room temperature (RT). Then, the first primary antibodies were incubated overnight at 4°C. After washing in PBST, added the secondary antibody anti‐Mouse/Rabbit Alexa Fluor 555 or 594 or 488 dye conjugated and incubated for 2 h. The antigen was repaired once again in the EDTA antigen retrieval buffer (pH 8.0) by the microwave oven to prepare for the next primary antibody, and the staining process was the same as the first cycle. After all the staining cycles finished, slides were washed in PBS, and then incubated with 4′,6‐diamidino‐2‐phenylindole (DAPI) at RT for 15 min. Images were captured by fluorescent microscopy in a dark place. Primary antibodies used for IHC are listed in Table S1.

Positively stained cells for CD8, PD1 and CD74 in each 1‐mm‐diameter TMA core were counted manually by two independent pathologists under high‐power magnification (200×), respectively. The mean number was set as the cutoff value for PD1, CD8, LSD1 and CD74 expression. PD‐L1 positive staining was determined as described previously.^21^ More than 5% positive area of PD‐L1 expression in tumour cells was defined as positive.

### 2.3 Cell lines, cell culture and transfection

Two mouse HCC cell lines h22 cells and hepa1‐6 cells (purchased from the Chinese Academy of Sciences Shanghai Branch Cell Bank) were used in this study. In brief, all the cells were cultured in Dulbecco's Modified Eagle Medium (DMEM; Invitrogen) with 10% foetal bovine serum (FBS; Invitrogen) and incubated at 37°C in a humidified atmosphere containing 5% CO_2_.

LSD1‐shRNA expression lentiviral vectors were purchased from Shanghai GeneChem Co., and the lentiviral vector was transfected according to the manufacturer's instructions. Sequences of interference used for shRNA are listed in Table S2. Transfected cells were selected using puromycin for 7 days prior to assay. OG‐L002 (purchased from APExBIO), an inhibitor of LSD1, was used as 50 µM for 48 h to inhibit the bioactivity of LSD1 in h22 or hepa1‐6 cells.

### 2.4 Quantitative real‐time polymerase chain reaction, western blotting and immunofluorescence assay

Quantitative real‐time polymerase chain reaction (*q*RT‐PCR) was performed on the QuantStudio3 Real‐Time PCR System. Glyceraldehyde 3‐phosphate dehydrogenase (GAPDH) was used as an internal control. The relative expression was analysed by the comparative cycle threshold (Ct) method, according to the equation 2^−(ΔCt_Experimental_group‐ ΔCt_Control_group)^. Sequences (5′‐3′) of primers used for *q*PCR are listed in Table S2. All experiments were performed in triplicate.

Western blotting was done as described previously.^22^ Primary antibodies used are listed in Table S1. Tubulin was used as an internal control.

Immunofluorescence assay was performed as previously described.^22^ Primary antibodies used are listed in Table S1. The slices were examined using fluorescence microscopy (Leica Microsystems Imaging Solutions).

### 2.5 Isolation and culture of peripheral blood mononuclear cells from mice

Peripheral blood mononuclear cells (PBMCs) were obtained as described in the Supplementary Materials and Methods. Fifty micromolars OG‐L002 for 48 h was used in the study and Dimethyl sulfoxide (DMSO) was used as negative control.

### 2.6 Enzyme‐linked immunosorbent assay

PBMCs isolated from mice were cultured for 72 h, and then the supernatant was used for IFN‐γ and Granzyme B (Gzmb) enzyme‐linked immunosorbent assay (ELISA) following the manufacturer's protocol (ELISA kits were purchased from R&D and ElabScience).

### 2.7 mRNA sequencing (RNA‐seq) and chromatin immunoprecipitation sequencing

PBMCs isolated from mice, h22/hepa1‐6 cells and tumour tissue from mouse model were used for RNA‐seq as in our previous study.^23^ In brief, after total RNA was isolated using the TRIzol reagent, RNA‐seq library preparation was carried out according to the manufacturer's guidelines (Illumina).

RNA‐seq data were analysed in detail as described in Supplementary Materials and Methods. PBMCs isolated from mice and h22/hepa1‐6 cells were used for chromatin immunoprecipitation sequencing (ChIP‐seq) analysis immunoprecipitated by anti‐LSD1 antibody. Precipitated DNA samples were prepared for deep sequencing according to the manufacturer's guidelines (Illumina).

### 2.8 Chromatin immunoprecipitation followed by quantitative PCR

PBMCs isolated from mice were cultured to 90% confluence in 10 cm dishes and crosslinked with 1% formaldehyde for 10 min at RT. Crosslinking was terminated by adding glycine to a final concentration of .125 mol/L, followed by washing with PBS. Cells were harvested, scraped and stored at −80°C.

For ChIP, cells were lysed in a lysis buffer containing protease inhibitors, and chromatin was fragmented by sonication. The lysate was incubated overnight at 4°C with antibodies against H3K4me1 (Table S1) in combination with Protein A+G magnetic beads (Magna ChIP, Cat. No. 16–663, PERFEMIKER). After washing, the chromatin was eluted and incubated with Proteinase K at 65°C overnight to reverse crosslinks. DNA was purified by phenol‐chloroform extraction and treated with RNase A (Thermo Scientific, Cat. No. 01000913).

qPCR was performed using AceQ qPCR SYBR Green Master Mix (GenScript, Cat. No. Q121‐02) on a CFX96 Real‐Time PCR Detection System (Bio‐Rad). The reaction mixture (10 µL) contained 5 µL of 2×SYBR Green Mix, .5 µL of each primer (10 µM), 2 µL of template DNA and ddH₂O to 10 µL. Cycling conditions were: 95°C for 5 min, followed by 40 cycles of 95°C for 10 s and 60°C for 30 s. Primers were designed based on predicted binding sites of H3K4me1 in the promoter regions of Gzmb and Cd28 (Table S3).

### 2.9 Single‐cell mRNA sequencing

Tumour tissues were collected and processed within 90 min after surgery. For the quality check and counting of single‐cell suspension, the cell survival rate is generally above 85%. The cells that have passed the test are washed and resuspended to prepare a suitable cell concentration of 700∼1200 cells/µL for 10x Genomics Chromium. The system is operated on the machine. GEMs (Gel Bead in Emulsion) were constructed for single‐cell separation according to the number of cells to be harvested. After GEMs were normally formed, GEMs were collected for reverse transcription in a PCR machine for labelling. The GEMs were oil‐treated, and the amplified cDNA was purified by magnetic beads, and then subjected to cDNA amplification and quality inspection. The 3ʹ Gene Expression Library was constructed with the quality‐qualified cDNA. After fragmentation, adaptor ligation, sample index PCR and so on, the library is finally quantitatively examined. The final library pool was sequenced on the Illumina Novaseq 6000 instrument using 150‐base‐pair paired‐end reads. Single‐cell mRNA sequencing (scRNA‐Seq) data were analysed in detail as described in Supplementary Materials and Methods.

### 2.10 Proliferation assay and apoptosis assay

CCK‐8 and PI‐Annexin V were used to evaluate the proliferation and apoptosis of tumour cells as in our previous study.^24^

### 2.11 Tumour growth assay in multiple mouse models

Male C57BL/6 mice (6 weeks old, purchased from Beijing Vital River Laboratory Animal Technology Co., Ltd) were used in this study. Three models were constructed to mimic different scenarios (Figure 5 and Figure S3). Model 1: 5×10^6^ h22 cells in .1 mL of DMEM were injected subcutaneously in the flank of mice. Each group includes six mice and experimental groups include as follows: Group 1: Negative Control (.85% saline); Group 2: 10 mg/kg rat anti‐mouse PD1 antibody (HRP00262‐012, .85% saline as vehicle, provided as gifts by Hengrui Medicine Com.) was intraperitoneally injected for six times (three times every week); Group 3: 20 mg/kg OG‐L002 was intraperitoneally injected once a day for 14 days; Group 4: 10 mg/kg rat anti‐mouse PD1 antibody plus 20 mg/kg OG‐L002 were administered. Model 2: 1×1 mm^3^ recurrent xenograft obtained from Group 2 in Model 1 was used to subcutaneously transplant into a new mouse as Model 2 as described previously.^24^ The experimental group and administration were the same as the Model 1. Model 3: recurrent xenograft mice models were obtained from Group 2 in Model 1. Recurrent xenografts were completely removed from the mice before tumour grew to 2 cm in diameter. 1×1 mm^3^ recurrent xenograft was obtained and then re‐transplanted in the contralateral subcutaneous of the same mice. The experimental groups (Negative Control and anti‐mouse PD1 antibody plus OG‐L002) were administrated the same as Model 1 did. Experimental drugs were administered when xenografts were palpable. Tumour volume was measured using a digital caliper and calculated as Volume = .5 × (Length × Width^2^/ 2). Tumour‐bearing mice were sacrificed before tumour grew to 2 cm in diameter. Mice without tumour growth were observed for 4 months after inoculation and sacrificed. All experiments were approved by the Animal Ethics Committee of Zhongshan Hospital, Fudan University.

### 2.12 Flow cytometry analysis

Xenografts and peripheral blood were harvested from mice treated with rat anti‐mouse PD1 antibody or/and OG‐L002. Flow cytometry was done as described previously.^22^, ^25^ Briefly, the suspension of tumour cell, tumour infiltrating lymphocyte (TIL) or immune cell were stained with primary antibodies as listed in Table S1. Flow cytometry was analysed by BD LSRFortessa (BD Biosciences), and data analysis was carried out using FlowJo 10.5.3 (Tree Star).

### 2.13 Statistical analysis

Statistical analyses were carried out using SPSS (20.0). Kaplan−Meier curves were used to represent survival, where significance was calculated with the log‐rank test. Significant differences in tumour growth were determined by an unpaired *t* or Mann−Whitney test. Significant differences in cell subsets were determined by an unpaired *t*‐test. Estimate of stained cells between para‐tumour tissues and tumour tissues was analysed by paired *t*‐test. Values of *p* < .05 were considered significant.

## 3 RESULTS

### 3.1 Expression and implication of LSD1/PD1/PD‐L1/CD8 in HCC patients

**FIGURE 1:**
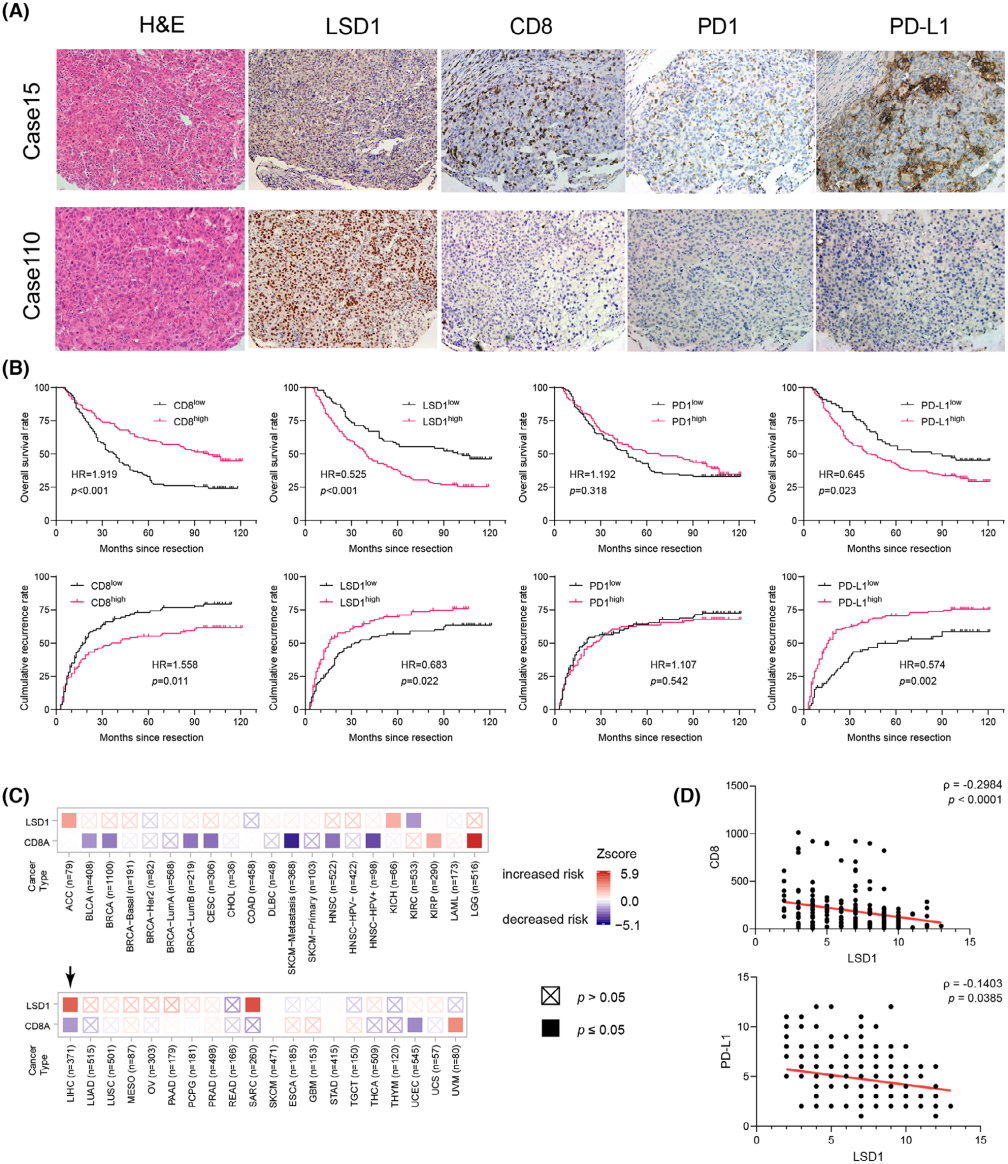
Expression and potential implication of LSD1, PD1, PD‐L1, CD8 in HCC patients. (A) Two representative HCC patients (Case 15 and Case 110) showing different levels of LSD1, CD8, PD1 and PD‐L1 expression (100×). Patient 1 shows LSD1^low^CD8^high^PD1^high^PD‐L1^high^ and Patient 2 shows LSD1^high^CD8^low^PD1^low^PD‐L1^low^. (B) Kaplan−Meier estimate of overall survival or cumulative recurrence in the whole cohort (*N* = 206) with different levels of CD8, LSD1, PD1 and PD‐L1(log‐rank test). (C) Modules exploring associations between gene expression and tumour outcome in TCGA: Gene Outcome module provides the clinical relevance of gene expression across various cancer types. The Cox proportional hazard model is used to evaluate the outcome significance of gene expression. Heatmap shows the normalized coefficient of the gene LSD1 and CD8A in Cox model. LIHC (liver hepatocellular carcinoma) is the only cancer type that LSD1 increases risk and CD8A decreases risk significantly across all the 41 cancer types in TCGA. (D) The correlations between LSD1 expression and CD8 (ρ = −.2984, *p* < .0001) or PD‐L1 (ρ = −.1403, *p* = .0385) expression were calculated on protein level in HCC cohort (*N* = 206) and PD1‐treated‐HCC cohort (*N* = 12) using Spearman's rank correlation test.

Evading immune surveillance is a crucial hallmark of cancer, wherein the TME plays a pivotal role in influencing tumorigenesis, metastasis and therapeutic response.^26^ Notably, the expression of PD‐L1 on tumour cells enables evasion from CD8^+^ T cell attacks and has emerged as a predictive indicator for assessing tumour response to PD1 antibody therapy.^10^ Preliminary evidence suggests that LSD1 plays a regulatory role in the interaction between tumour cells and the TME.^11^ Consistent with our prior study,^27^, ^28^ PD‐L1 expression was predominantly observed in the tumour cell membrane, infiltrating lymphocytes and endothelial cells (Figure 1A). Based on a predefined cut‐off value of 5% positive expression in tumour cells,^29^ HCC patients were categorized into PD‐L1‐positive (high expression) and PD‐L1‐negative (low expression) subgroups. Survival analysis revealed that PD‐L1‐positive HCC patients exhibited significantly poorer prognosis in terms of shorter overall survival (OS) and a higher cumulative recurrence rate (Figure 1B). Subsequently, we explored the expression of PD1 in HCC tumour tissues, with positive expression predominantly detected in TILs and endothelial cells (Figure 1A). However, PD1 expression did not correlate with OS or cumulative recurrence rate in HCC patients (Figure 1B). Cytotoxic CD8^+^ T cells in tumour tissues, often considered a favourable factor against tumour development and progression,^5^ were quantified through IHC staining. The mean number of cytotoxic CD8^+^ T cells in tumour samples was 192.31 ± 14.07, and a greater number of CD8^+^ T cells were strongly associated with longer OS and a lower cumulative recurrence rate (Figure 1A,B). Positive staining for LSD1 was localized in the nucleus of tumour cells and some infiltrating lymphocytes (Figure 1A). A subsequent semi‐quantitative analysis of LSD1 expression was performed. HCC patients with high LSD1 levels in tumour samples experienced shorter OS and a higher cumulative recurrence rate than those with low LSD1 expression (Figure 1B). In silico analysis of The Cancer Genome Atlas (TCGA) data highlighted a unique association in HCC that high LSD1 and low CD8A levels coincided with an elevated risk of death among all 41 cancer types (Figure 1C). In the TMAs, our results showed that HCC patients expressing high levels of LSD1 protein tended to have low PD‐L1 protein expression in tumour cells (ρ = −.1403, *p* = .0385) and a reduced number of CD8^+^ T cells (ρ = −.2984, *p* < .0001) (Figure 1D). These findings suggest that LSD1 may serve as a dual regulator, influencing PD‐L1 expression in tumour cells and the infiltration of CD8^+^ T cells in the TME, ultimately determining sensitivity to ICB therapy.

### 3.2 Inhibition of LSD1 impairs the growth of tumour cells and enhances the PD‐L1 expression in liver cancer cells in vitro

**FIGURE 2:**
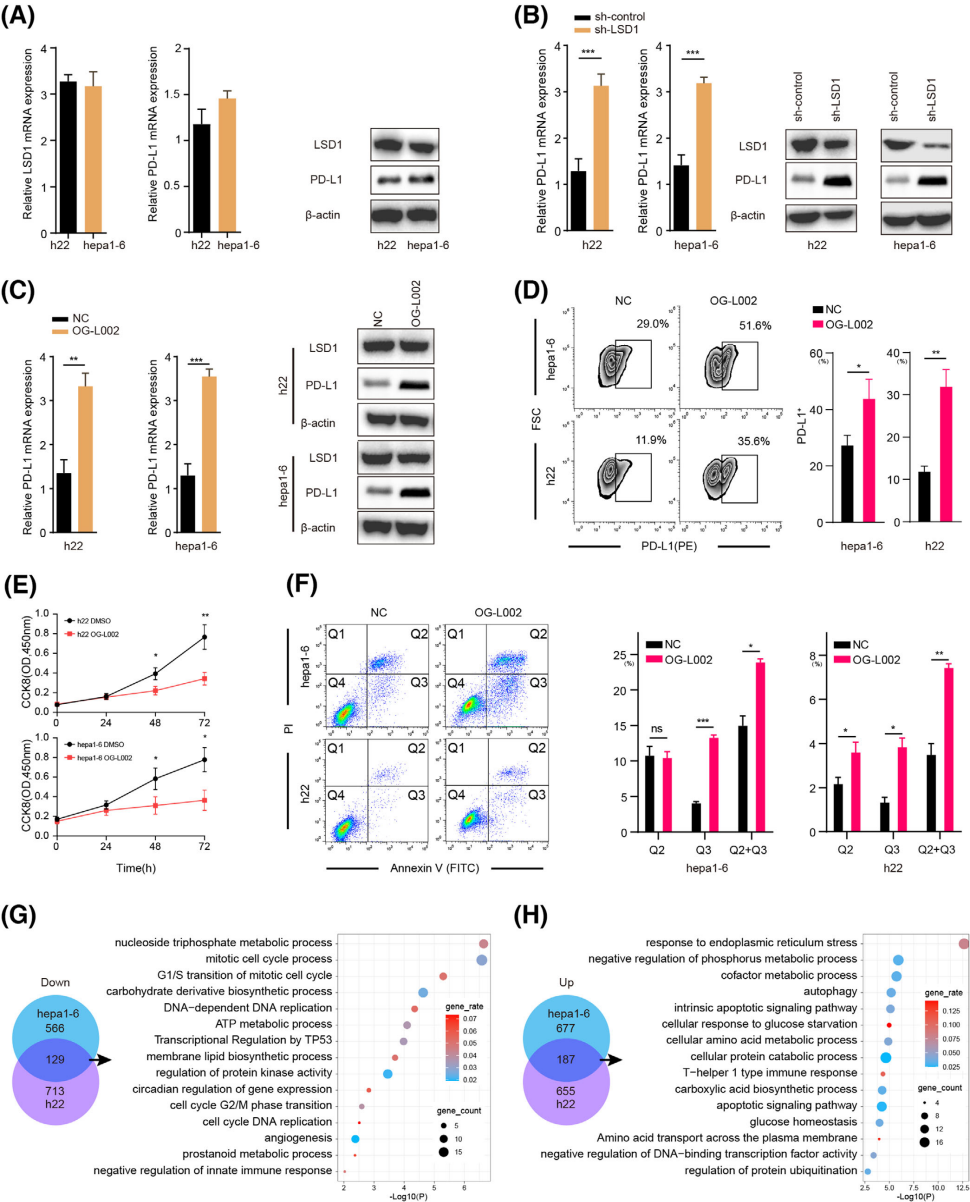
Inhibition of LSD1 increases PD‐L1 expression in liver cancer cells. (A) qRT‐PCR and western blot analysis of LSD1 and PD‐L1 expression in two mice HCC cell lines h22 and hepa1‐6 (presented as mean ± SD). (B) Relative mRNA and protein level of PD‐L1 in h22 and hepa1‐6 cell line after LSD1 shRNA incubation (presented as mean ± SD, ***: *p *< .001, *t*‐test). (C) Relative mRNA level of PD‐L1 and protein level of PD‐L1 and LSD1 in h22 and hepa1‐6 cell lines after LSD1 inhibitor OG‐L002 incubation for 48 h (presented as mean ± SD, ***: *p *< .001, *t*‐test). (D) FACS analysis showed the proportion of PD‐L1^+^ cells in h22 and hepa1‐6 cell lines after LSD1 inhibitor OG‐L002 incubation for 48 h (presented as mean ± SD, *: *p *< .05, **: *p *< .01, *t*‐test). (E) Cell viability of h22 or hepa1‐6 were assessed after OG‐L002 incubation for 0, 24, 48 and 72 h using CCK8 assay (presented as mean ± SD, *: *p* < .05, **: *p* < .01, *t*‐test). (F) Flow cytometry showed apoptosis of h22 and hepa1‐6 after LSD1 inhibitor OG‐L002 incubation for 48 h. Annexin V and PI staining was used to distinguish early apoptotic (Q3, Annexin V^+^PI^−^) and later apoptotic cells (Q2, Annexin V^+^PI^+^). The proportion of early apoptotic cells (Q3), later apoptotic cells (Q2) and all the apoptotic cells (Q2+Q3) were compared between negative control group (NC) and the OG‐L002 use group (presented as mean ± SD, *: *p* < .05, **: *p* < .01, ***: *p* < .001, one‐way ANOVA test, Tukey's multiple comparisons test for multiple comparison). (G, H) The overlapped differentiated genes were selected from hepa1‐6 and h22 cells after OG‐L002 incubation for 48 h. The bubble map showed enriched terms of differentiated genes.

To further elucidate the relationship between LSD1 and PD‐L1 expression in tumour cells, we initially assayed the expression of LSD1 and PD‐L1 in two mouse HCC cell lines, h22 and hepa1‐6, using *q*RT‐PCR and western blotting (Figure 2A). Subsequently, we successfully constructed HCC cells with stable knockdown of LSD1 expression, as confirmed by *q*RT‐PCR, western blotting and immunofluorescence (Figure 2B and Figure S1). Remarkably, the knockdown of LSD1 expression in HCC cells led to a significant increase in PD‐L1 expression (Figure 2B). Based on these results, we chose 50 µM OG‐L002 for 48 h to do further function assays in vitro. We next examined the role of OG‐L002 in the expression of PD‐L1 of tumour cells and found that LSD1 inhibitor significantly upregulated the expression of PD‐L1 without affecting the protein level of LSD1 (Figure 2C). Flow cytometry analysis further confirmed that the LSD1 inhibitor significantly increased the proportion of PD‐L1^+^ HCC cells (Figure 2D).

Additionally, we explored the impact of LSD1 on the functional aspects of tumour cells. The LSD1 inhibitor substantially suppressed the proliferation of liver cancer cells and induced apoptosis (Figure 2E,F). To gain insights into the molecular mechanisms underlying the influence of LSD1 in liver cancer cells, we performed RNA‐seq analysis. Our data revealed that the mRNA levels of 695 and 842 genes were significantly downregulated, while 864 and 842 genes were upregulated in hepa1‐6 cells and h22 cells after treatment with LSD1 inhibitor, respectively (Figure 2G,H, Tables S4 and S5). These overlapped differentiated genes, including 129 downregulated and 187 upregulated genes, were mainly associated with cell cycle processes, apoptotic regulation and ATP metabolic processes in liver cancer cells (Figure 2G,H). Collectively, these findings suggest that LSD1 deletion inhibits tumour growth by suppressing the cell cycle and promoting apoptotic signals, while concurrently upregulating PD‐L1 expression in tumour cells.

### 3.3 LSD1 deletion rejuvenates effector CD8^+^ T cells and enhances anti‐tumour immunity in vitro

**FIGURE 3:**
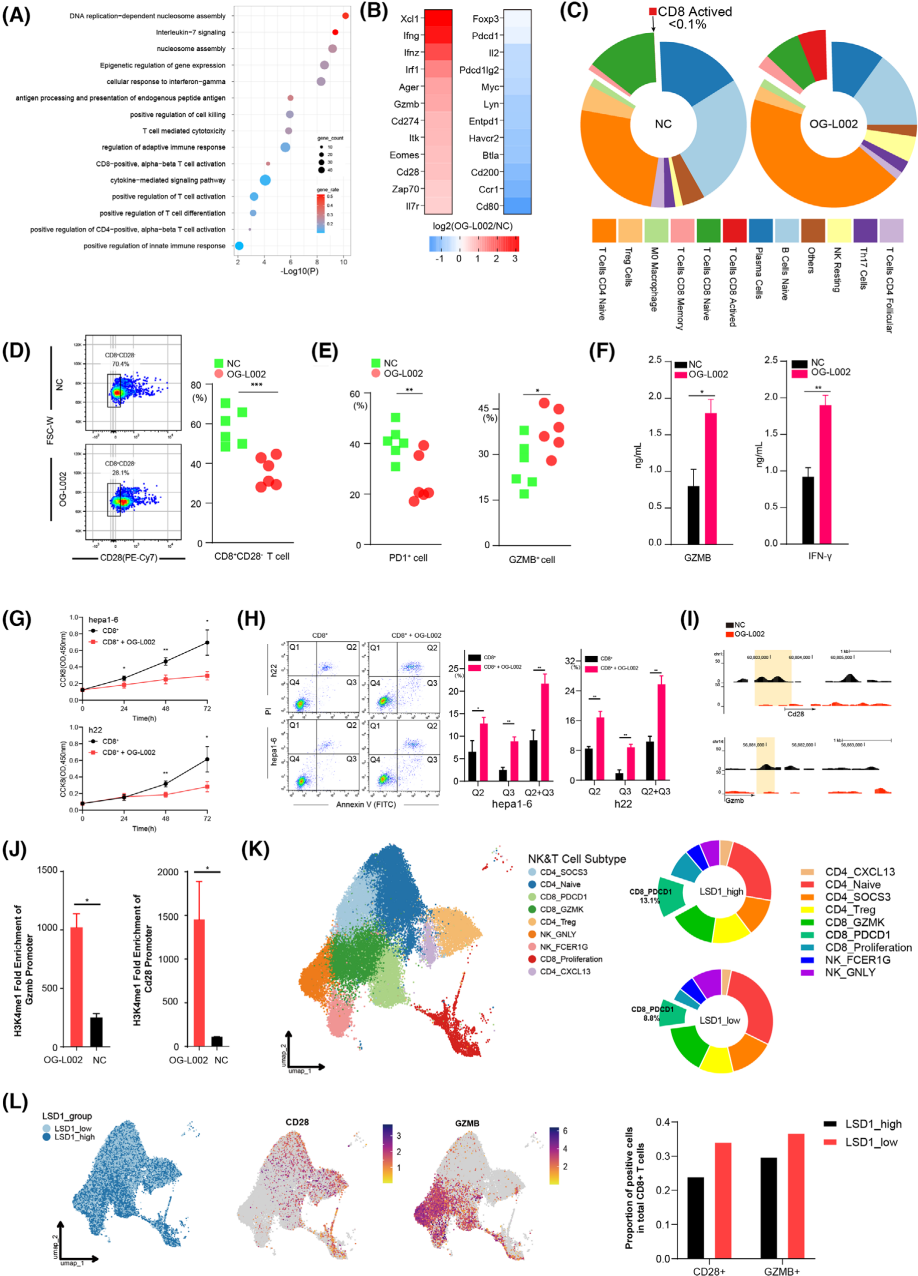
LSD1 inhibitor reshapes PBMC environment and rejuvenates effector CD8^+^ T cells. (A) Upregulated differentiated genes were selected from PBMC after OG‐L002 incubation for 48 h, and the bubble map showed enriched terms of these differentiated genes. (B) Heat map showed representative genes including cytotoxic molecules, co‐stimulatory molecules and inflammatory response‐related genes were changed in PBMC after OG‐L002 incubation for 48 h. (C) Proportion changes of different immune cells in PBMC (the proportions are calculated based on RNA‐seq data and employed the algorithm method CIBERSORT). CD8 T cells are largely activated from the naïve after OG‐L002 use. ‘Others’: Sum of the immune cells whose proportion is less than 1.5% (neither in OG‐L002 group or NC group), including mast cells, neutrophil cells, eosinophil cells, B cells memory, M1 macrophage, M2 macrophage, T cells CD4 memory, Th1 cells, Th2 cells, monocyte, gamma delta T cells, NK active, DC active and DC immature. (D) Flow cytometry analysis results showed the proportion of CD28^−^CD8^+^ senescent T cells in CD8^+^ T cells after treated with OG‐L002 in comparison to the control (NC) group. Each experimental group consisted of two samples, and the analysis was conducted with three replicates (***: *p *< .001, *t*‐test). (E) Flow cytometry analysis results showed the proportion of PD1^+^ T cells and GZMB^+^ T cells in CD8^+^ T cells after treated with OG‐L002 in comparison to the control (NC) group. Each experimental group consisted of two samples, and the analysis was conducted with three replicates (ns: not significant, *: *p* < .05, **: *p* < .01, *t*‐test). (F) CD8^+^ cells from PBMC were isolated to perform further experiment. ELISA showed concertation of GZMB and IFN‐γ in the supernatants of CD8^+^ T cells treated with OG‐L002(*: *p* < .05, **: *p* < .01, *t*‐test). (G) Cell viability of h22 or hepa1‐6 were assessed for 0, 24, 48 and 72 h using CCK8 assay after co‐cultured with supernatants of CD8^+^ T cells pretreated with LSD1 inhibitor (presented as mean ± SD, *: *p* < .05, **: *p* < .01, *t*‐test). (H) Flow cytometry showed apoptosis of h22 and hepa1‐6 after co‐cultured with supernatants of CD8^+^ T cells pretreated with LSD1 inhibitor. Annexin V and PI staining was used to distinguish early apoptotic (Q3, Annexin V^+^PI^−^) and later apoptotic cells (Q2, Annexin V^+^PI^+^). The proportion of early apoptotic cells (Q3), later apoptotic cells (Q2) and all the apoptotic cells (Q2+Q3) were compared between negative control group (NC) and the OG‐L002 use group (presented as mean ± SD, *: *p* < .05, **: *p* < .01, one‐way ANOVA test, Tukey's multiple comparisons test for multiple comparison). (I) LSD1 ChIP‐Seq peaks at representative genes that may influence the function of immunity. (J) H3K4me1‐fold enrichment at the promoter regions of Gzmb and Cd28 genes (presented as mean ± SD, *: *p* < .05, *t*‐test). (K) Uniform Manifold Approximation and Projection (UMAP) plot depicted distinct NK and T cell clusters from scRNA‐Seq data of eight HCC patients. Pie charts illustrated the proportional distribution of various cell clusters between the LSD1 high‐ and low‐expression groups. (L) UMAP plot showed the distribution of cells from LSD1 high/low groups. Feature plots displayed the distribution of CD28^+^ and GZMB^+^ cells across all NK and T cells. A bar plot on the right showed the average proportions of CD28^+^ and GZMB^+^ cells within total CD8^+^ T cells between LSD1 high/low‐expression groups.

To further investigate the impact of LSD1 on the immune microenvironment, PBMCs were isolated from mouse spleens and treated with 50 µM OG‐L002 for 48 h. Subsequent RNA‐seq and ChIP‐seq were performed. RNA‐seq showed 3807 differentiated genes (including 1387 upregulated genes and 2420 downregulated genes) in LSD1 inhibitor‐treated PBMCs compared to the control (Table S6). Gene ontology analysis unveiled that these differentially expressed genes were mainly associated with the positive regulation of T cell activation, positive regulation of T cell differentiation, antigen processing and presentation of endogenous peptide antigens, positive regulation of innate immune response and cell response to IFN‐gamma (Figure 3A). Among these differentiated genes, cytotoxic molecules and co‐stimulatory molecules, such as CD28, GZMB, IFN‐γ(ifng), IFN‐z (ifnz) and immune checkpoint molecule PD‐L1 (CD274), exhibited significant upregulation, while inflammatory response‐related genes such as Btla, CD39 (Entpd1), PD1 (pdcd1) and Foxp3 were notably downregulated following LSD1 inhibitor treatment (Figure 3B). GZMB, secreted by CD8^+^ cytotoxic T lymphocytes (CTLs), induces tumour cell apoptosis,^30^, ^31^ and co‐stimulatory molecule CD28 is crucial for the proliferation, activation, cytokine production and development of CD8^+^ T cells.^32^ These results suggest that LSD1 inhibitor likely reshapes the anti‐tumoral immune environment. To identify the specific immune cell populations influenced by the LSD1 inhibitor, we employed the CIBERSORT algorithm^33^, ^34^ to calculate the abundance of different immune cells based on the RNA‐seq data. The results showed an increased proportion of effector CD8^+^ cells, CD4^+^ naive T cells and Natural killer (NK) resting cells, alongside a modest decreased proportion of Treg cells, naive B cells and plasma cells after treatment with LSD1 inhibitor (Figure 3C). Subsequent analysis of the CD8^+^ T cell subpopulations in PBMCs demonstrated significant increased proportion in active CD8^+^ T cells (increased from < .1% to 5.5%) and memory CD8^+^ T cells (increased from 12.1% to 27.1%), coupled with decreased proportion of naive CD8^+^ T cells (decreased from 13.7% to 7.1%) in PBMCs following treatment with the LSD1 inhibitor (Figure 3C). These data indicate that LSD1 deletion rejuvenates CD8^+^ T cells.

Flow cytometry analysis corroborated the aforementioned findings. CD28, a co‐stimulatory molecule, holds a crucial position in promoting the proliferation, activation, cytokine production and maturation of CD8^+^ T cells. Notably, the reduced expression of CD28 is acknowledged as a distinctive feature of senescent T cells.^35^ The proportion of CD28^−^CD8^+^ senescent T cells in CD8^+^ T cells treated with OG‐L002 was reduced to 34.9% ± 6.6%, compared to that of the control (57.5% ± 8.2%) (Figure 3D). Functionally, the proportion of PD1^+^ exhausted T cells in CD8^+^ T cells treated with OG‐L002 was 20.6% ± 8.5%, significantly lower than that of the control (40.6% ± 5.9%) (Figure 3E). Meanwhile, the proportion of GZMB^+^ activated T cells in CD8^+^ T cells notably increased (37.5% ± 6.5% vs. 25.5% ± 7.2%) after treatment with OG‐L002 (Figure 3E). The isolation of CD8^+^ cells from PBMCs was performed to further experiments. ELISA showed the increased concertation of GZMB and IFN‐γ in the supernatants of CD8^+^ T cells treated with LSD1 inhibitor compared to the control (Figure 3F). Even more importantly, when liver cancer cells were cultured with the supernatants of CD8^+^ T cells pretreated with the LSD1 inhibitor, the proliferation ability of the cancer cells was significantly inhibited, and the apoptosis rate was enhanced (Figure 3G,H). To elucidate the molecular mechanism underlying LSD1‐mediated rejuvenation of CD8^+^ T cells, LSD1 ChIP‐seq revealed significantly altered peaks of genes related to the activation of CD8^+^ T cells in the LSD1 inhibitor‐treated group. Significantly altered expressions of effector‐associated genes, namely cd28 and gzmb, were observed in PBMCs after OG‐L002 treatment. Notably, a discernible reduction in LSD1 enrichment within the promoter region was detected after OG‐L002 treatment in comparison to the control group (Figure 3I). We further conducted chromatin immunoprecipitation followed by quantitative PCR experiments, which confirmed that treatment with OG‐L002 significantly increased the enrichment of H3K4me1 upstream of the cd28 and gzmb genes in PBMCs (Figure 3J).

Although in vitro experiments have shown that LSD1 inhibition can modulate immune cells and restore CD8^+^ T cell function through epigenetic regulation, thereby enhancing anti‐tumour capabilities, we sought to explore whether a similar phenotype exists in TILs within the TME. To this end, we performed scRNA‐Seq on surgical tumour samples from eight HCC patients and conducted a detailed analysis of the NK and T cell subpopulations (Figure S2). The results revealed that, compared to the LSD1 high expression group, the LSD1 low expression group exhibited an upregulation in the proportion of CD4_Naïve and CD8_GZMK cells (29.26% vs. 23.46%, 15.73% vs. 14.60%, respectively), and a downregulation in the proportion of CD4_Treg and CD8_Proliferation cells (10.73% vs. 12.68%, 3.96% vs. 8.73%, respectively). Notably, the CD8_PDCD1 subpopulation in the LSD1 low expression group represented only 8.84%, while in the LSD1 high expression group, CD8^+^ T cells expressing exhaustion markers accounted for 13.08% of the TME (Figure 3K). Additionally, UMAP analysis revealed a higher distribution of CD28^+^ and GZMB^+^ cells in the LSD1 low‐expression group compared to the LSD1 high‐expression group. When we calculated the proportion of CD28^+^ and GZMB^+^ cells among all CD8^+^ cells, the LSD1 low‐expression group showed a higher average proportion of these cells than the high‐expression group (Figure 3L). Through scRNA‐Seq analysis, we found that TILs within the TME exhibited nearly identical phenotypes to those observed in PBMCs in vitro. These findings suggest that LSD1 acts as an epigenetic switch, regulating the ‘off‐on’ transition for the rejuvenation of CD8^+^ T cells and remodelling the anti‐tumoral immune microenvironment.

### 3.4 Inhibition of LSD1 suppresses tumour growth and remodels anti‐tumoral niches in vivo

**FIGURE 4:**
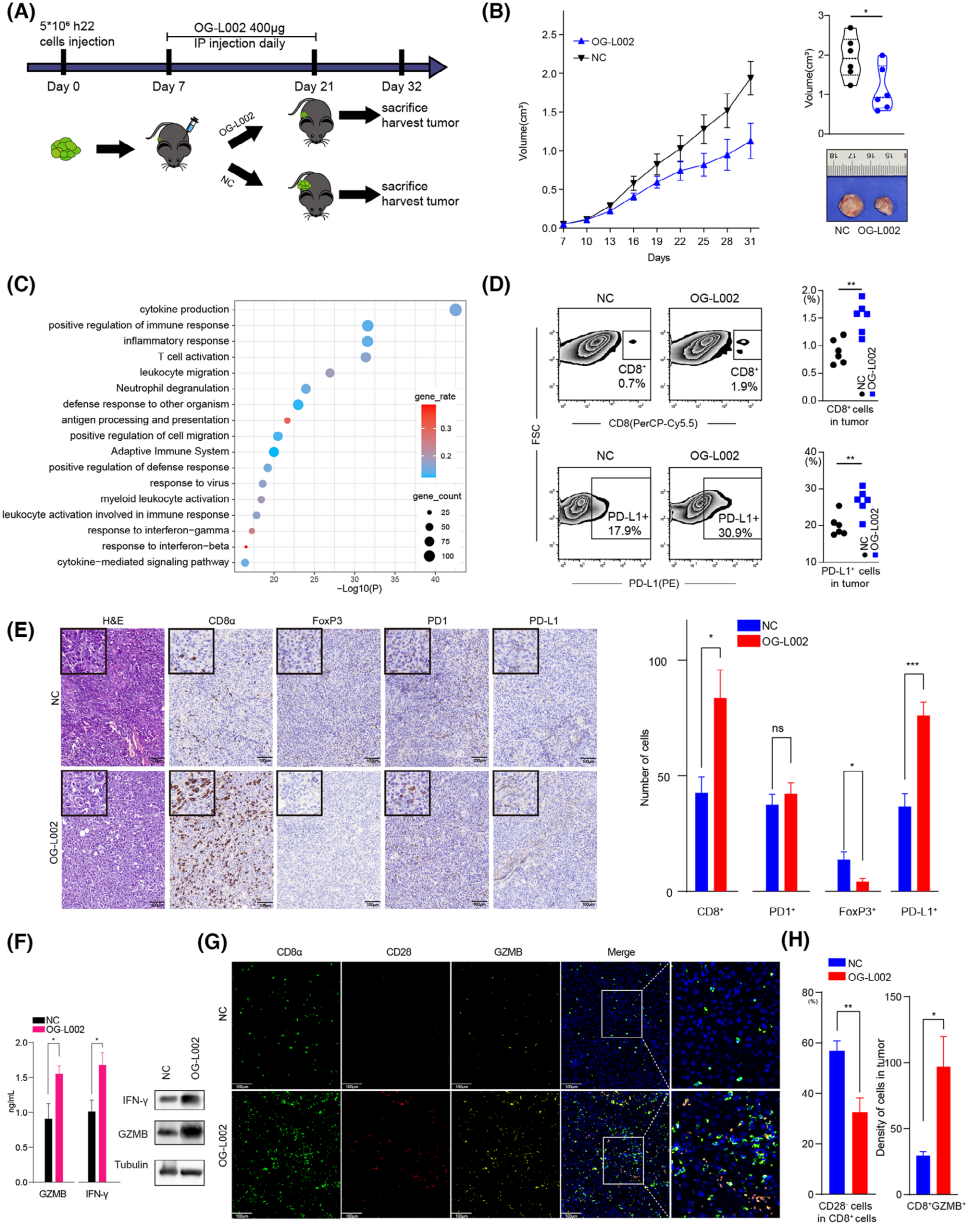
Inhibition of LSD1 suppresses tumour progression in liver cancer model. (A) Schematic of experimental design: 5×10^6^ h22 cells in .1 mL of DMEM were injected subcutaneously in the flank of mice on Day 0. Four hundred micrograms OG‐L002 was intraperitoneally (IP) injected once a day for 14 days since Day 7. Peripheral blood or tumours were harvested on Day 32. (B) Tumour growth volume over time following intraperitoneal injection of OG‐L002 or injected with .85% saline (*n* = 6 for each group. *: *p* < .05 *t*‐test; error bars represent SEM). Tumours were harvested and calculated their volume after mice sacrificed, and representative tumours from different groups were shown in the picture (*: *p* < .05, *t*‐test). (C) A bubble chart of top 20 terms in enrichment analysis of upregulated genes (log_2_(OG‐L002 group/control group) > .58) in OG‐L002 group versus control group. (D) Flow cytometry indicated proportion of CD8^+^ T cells and PD‐L1^+^ cells in the tumour microenvironment from different groups (*n* = 6 for each group, **: *p* < .01, *t*‐test). (E) Representative IHC pictures of H&E, CD8, FoxP3, PD1 and PD‐L1 in harvested tumour tissues. The statistic results of number of CTL cells, Treg cells, PD1^+^ cells and PD‐L1^+^ cells in tumour tissues between LSD1 inhibitor group and the control group (presented as mean ± SD, ns: not significant, *: *p* < .05, *t*‐test was used). (F) ELISA (left) and western blotting (right) analysis showed the expression of IFN‐γ and GZMB in peripheral blood CD8^+^ T cell (presented as mean ± SD, *: *p* < .05, **: *p* < .01, *t*‐test). (G, H) Representative picture showed multiple‐IHC staining CD8, CD28, GZMB and DAPI from OG‐L002 group and Negative Control group (NC). The statistic results of CD28^−^CD8^+^ senescent T cells and CD8^+^GZMB^+^ cells were shown on the right (presented as mean ± SD, ns: not significant, *: *p* < .05, **: *p* < .01, *t*‐test).

To further explore the effects of LSD1 inhibition on tumour growth and tumour immune microenvironment in vivo, we constructed a mouse model by subcutaneously injecting with 5×10^6^ h22 cells (Figure 4A). LSD1 inhibitor OG‐L002 significantly impeded the tumour growth of h22 cells (Figure 4B). Subsequently, RNA‐seq analysis of the harvested tumours revealed 880 differentiated genes (including 810 upregulated genes and 70 downregulated genes) in the OG‐L002‐treated xenografts compared to the control (Table S7). Enrichment analysis indicated that these upregulated genes were predominantly associated with inflammatory response, T cell activation, positive regulation of immune response, and antigen processing and presentation (Figure 4C). Flow cytometry analysis further revealed an increased proportion of CD8^+^ T cells in the LSD1 inhibitor‐treated tumours (Figure 4D). Additionally, the proportion of PD‐L1^+^ tumour cells in the xenografts from the LSD1 inhibitor‐treated group significantly surpassed that of the control group (Figure 4D). IHC staining also demonstrated that LSD1 inhibition resulted in an augmented number of CD8^+^ CTLs, enhanced expression of PD‐L1 and a reduced number of FoxP3^+^ Treg cells in tumour tissues (Figure 4E). Notably, LSD1 inhibition did not impact the number of intra‐tumoral PD1^+^ T cells (Figure 4E). Furthermore, ELISA and western blotting also showed increased levels of effector molecules, such as GZMB and IFN‐γ of CD8^+^ T cells, in the serum from OG‐L002‐treated group (Figure 4F). Multiple IHC staining also showed a significantly decreased number of CD28^−^CD8^+^ senescent T cells in the OG‐L002‐treated group compared to the control group (Figure 4G,H). Importantly, there was an increased proportion of GZMB^+^CD8^+^ T cells among the total CD8^+^ T cells, which was detected in the OG‐L002‐treated group compared to the control group (Figure 4G,H). These data comprehensively indicate that LSD1 inhibitor reshaped the anti‐tumoral immune microenvironment by rejuvenating of CD8^+^ T cells beyond merely suppressing tumour growth in vivo.

### 3.5 Combined therapy of LSD1 inhibitor and anti‐PD1 antibody blocks acquired resistance of PD1 inhibitor alone in vivo

**FIGURE 5:**
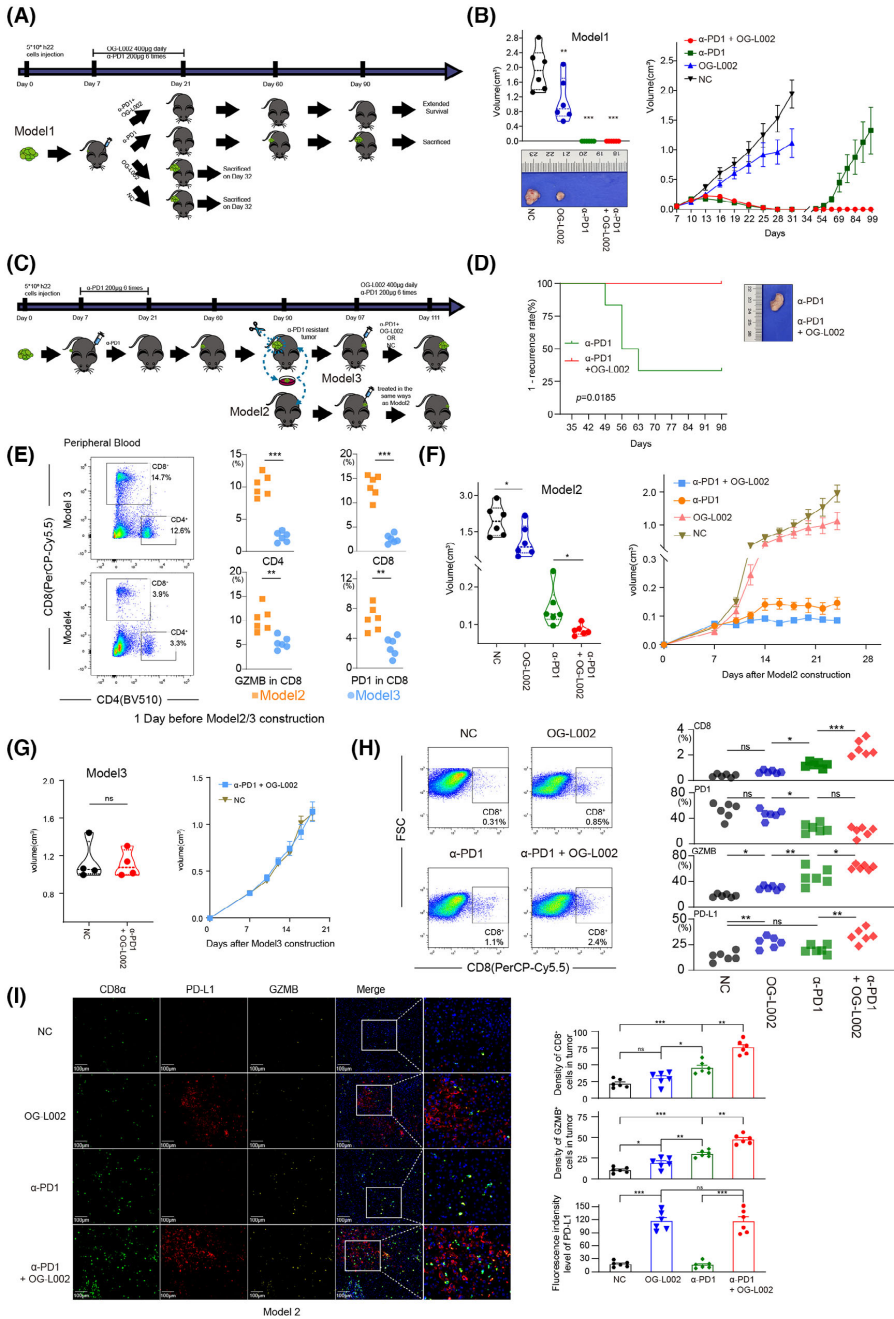
Combination of LSD1 inhibitor and PD1 blockage prevented from tumour recurrence after PD1 blockage alone. (A) Schematic of experimental design for Model 1: 5*10^6^ h22 cells in .1 mL of DMEM were injected subcutaneously in the flank of mice on Day 0. Four hundred micrograms OG‐L002 was intraperitoneally injected once a day for 14 days since Day 7. Two hundred micrograms rat anti‐mouse PD1 was intraperitoneally injected for six times (three times every week) since Day 7. Peripheral blood or tumours were harvested on Day 32 or the day the volume of recurrent tumour > 1.5 cm.^3^ (B) Tumour growth volume over time in Model 1 (*n* = 6 for each group, error bars represent SEM). Tumours were harvested and calculated the volume after mice sacrificed, and representative tumours from different groups were shown in the picture (*n* = 6 for each group, *: *p* < .05, **: *p* < .01, one‐way ANOVA, error bars represent SD). (C) Schematic of experimental design for Model 2 and Model 3: recurrent tumours from PD1 antibody treatment group in Model 1 were completely removed and transplanted to another new mice with same age (Model 2) or to the contralateral subcutaneous of the same mouse (Model 3). Then, the treatment of OG‐L002 or anti‐mouse PD1 was performed according to the same usage as Model 1. (D) Kaplan‒Meier analysis of tumour recurrence 35 days after Model 1 construction (log‐rank test). Representative tumour from anti‐PD1 group (α‐PD1) and combination use of anti‐PD1 and OG‐L002 group (α‐PD1 + OG‐L002) were shown in the picture. (E) Flow cytometry indicated proportion of CD8^+^ cells, CD4^+^ cells, PD1^+^ CD8^+^ cells and GZMB^+^ CD8^+^ cells of peripheral blood before Model 2 or Model 3 construction (*n* = 6 for each Model, **: *p* < .01, ***: *p *< .001, *t*‐test). (F) Tumour growth volume over time in Model 2 (*n* = 6 for each group, error bars represent SEM). Tumours were harvested and calculated their volumes after mice sacrificed (*n* = 6 for each group, *: *p* < .05, one‐way ANOVA, error bars represent SD). (G) Tumour growth volume over time in Model 3 (*n* = 4 for each group, error bars represent SEM). Tumours were harvested and calculated their volumes after mice sacrificed (*n* = 4 for each group, ns: not significant, *t*‐test). (H) Flow cytometry indicated proportion of CD8^+^ cells, PD‐L1^+^ cells, PD1^+^ CD8^+^ cells and GZMB^+^ CD8^+^ cells from tumour microenvironment in Model 2 (*n* = 6 for each group, ns: not significant, *: *p* < .05, **: *p* < .01, ***: *p *< .001, one‐way ANOVA). (I) Representative pictures showed multiple‐IHC staining CD8, PD‐L1, GZMB and DAPI in Model 2. The statistic results of GZMB^+^ cells, CD8^+^ cells and PD‐L1^+^ cells were shown on the right (*n* = 6 for each group, presented as mean ± SD, ns: not significant, *: *p* < .05, **: *p* < .01, ***: *p *< .001, one‐way ANOVA).

Shi^11^ previously reported that LSD1 inhibition could convert a ‘cold’ tumour (resistant to PD1 blockade) into a ‘hot’ tumour (sensitive to PD1 blockade) in a mouse melanoma model. In the present study, we sought to delve further into the roles of LSD1 inhibition and/or PD1 blockade within three mouse HCC models mimicking distinct TMEs. In Model 1, we subcutaneously inoculated with 5×10^6^ h22 cells (Figure 5A). We found that LSD1 inhibitor alone significantly repressed tumour growth of h22 cells compared to the control (Figures 4A and 5B). Interestingly, complete tumour regression was achieved in both the anti‐PD1 antibody alone group and combined therapy of LSD1 inhibitor and anti‐PD1 antibody group at the end of treatment (Figure 5B). Unexpectedly, tumour recurrence was observed in 66.7% (4/6) of the anti‐PD1 antibody‐treated group after discontinuation for more than 2 weeks, while no relapse occurred in the combined therapy group even 2 months after withdrawal of medication (Figure 5B,D). In order to further explore the response to PD1 inhibitor in different TME induced by LSD1 inhibitor and/or PD1 inhibitor, we constructed Model 2 and Model 3. In Model 2, we re‐transplanted 1×1 mm^3^ recurrent liver tumour derived from the anti‐PD1 antibody‐treated group of Model 1 into new C57BL/6 mice. After a palpable tumour was formed, these mice were classified into four sub‐groups and administered with different agents as described in Model 1 (Figure 5C). In Model 3, we radically remove the recurrent xenograft from tumour‐bearing mice treated with the anti‐PD1 antibody alone of Model 1 and re‐transplanted 1×1 mm^3^ recurrent tumour derived from the anti‐PD1 antibody‐treated group into the contralateral subcutaneous of the same mouse (Figure 5C). Before the construction of Model 2/3, the immune status in mice to be modelled was explored by Flow cytometry analysis. The results showed the peripheral blood in tumour‐bearing mice treated with anti‐PD1 antibody and then relapsed (anti‐PD1 antibody alone group in Model 1), manifested a compromised immune status (lower CD8^+^, lower CD4^+^ and lower GZMB^+^) before construction of Model 3 (Figure 5E). Conversely, the naïve C57BL/6 mice for the construction of Model 2 displayed a naturally anti‐tumour effector of immune status (higher CD8^+^, higher CD4^+^, higher GZMB^+^, but higher PD1^+^). In Model 2, anti‐PD1 antibody alone or LSD1 inhibitor alone exerted modest inhibitory effects on tumour growth of recurrent xenograft derived from the PD1 inhibitor‐treated group of Model 1 (Figure 5F). Strikingly, combined therapy of anti‐PD1 antibody and LSD1 inhibitor exhibited a more robust inhibition of tumour growth than either anti‐PD1 antibody alone or LSD1 inhibitor alone (Figure 5F). However, neither the anti‐PD1 antibody alone nor the combination therapy of LSD1 inhibitor and anti‐PD1 antibody achieved a complete response in Model 2, which was inconsistent with the outcome in Model 1 (Figure 5F). In Model 3, interestingly, combined therapy of anti‐PD1 antibody and LSD1 inhibitor did not significantly inhibit tumour growth (Figure 5G). Moreover, the velocity of tumour growth in Model 3 was faster than that in Model 1 (Figure 5B,G).

The tumours harvested from the four groups in Model 2 were then digested to perform Flow cytometry analysis or fixed to carry out multi‐IHC staining. Flow cytometry analysis showed that more GZMB^+^CD8^+^ cells and PD‐L1^+^ cells were found in the TME of the OG‐L002 group compared to the NC group, but OG‐L002 alone did not influence the number of CD8^+^ T cells or PD1^+^CD8^+^ T cells (Figure 5H). Meanwhile, the anti‐PD1 antibody‐treated group showed a higher number of CD8^+^ T cells than that of NC or OG‐L002 group, while the combined therapy of LSD1 inhibitor and anti‐PD1 antibody group showed the highest proportion of CD8^+^ T cells among all the four groups (Figure 5H). Importantly, the combined therapy group displayed a higher proportion of GZMB^+^ CD8^+^ T cells and PD‐L1^+^ tumour cells in TME compared to the anti‐PD1 antibody‐treated group, while the two groups showed no difference in PD1^+^CD8^+^ T cells (Figure 5H). Multi‐IHC staining further confirmed the differential numbers of positive cells for markers CD8, GZMB and PD‐L1 in TME from the four groups in Model 2 (Figure 5I). The decreased numbers of CD8^+^ and GZMB^+^ cells were observed in OG‐L002‐treated and NC group in Model 2 compared to those in Model 1, indicating that the recurrent xenografts exhibited the impaired effector T cell ability in TME compared with the original tumour (Figures 4G, 5G and Figure S4). These data in vivo underscore that the inhibitory role of anti‐PD1 antibody in tumour growth depended on the intricate interaction between tumour cells and the TME. LSD1 deletion likely creates a relapse‐free niche for HCCs in response to PD1 inhibitor therapy, leading to a longer DoR to PD1 inhibitor.

### 3.6 LSD1 inhibitor prevents from acquired resistance of PD1 blockage by remodelling relapse‐free niche and activating MHC class I/II‐dependent antigen processing and presentation in vivo

**FIGURE 6:**
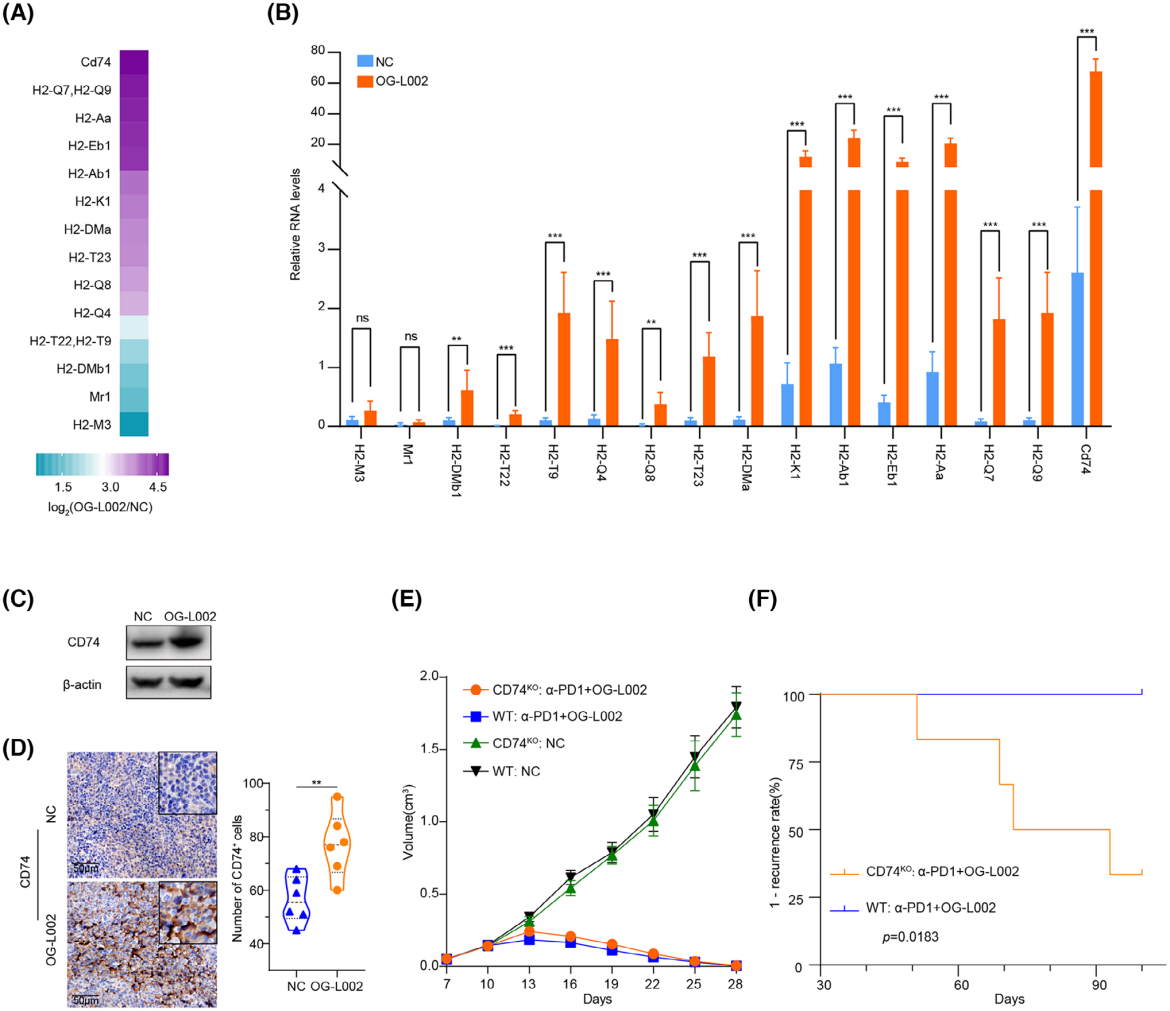
LSD1 inhibitor prevents from acquired resistance of PD1 blockage by remodelling relapse‐free niche and enhancing myeloid cell functions related to CD74 in vivo. (A) Heat map showed fold changes of histocompatibility‐associated genes in tumour tissues between the LSD1 inhibitor OG‐L002 group and the control group. (B) qRT‐PCR verified the copy number of the 16 histocompatibility‐associated genes selected from mRNA‐seq in tumour tissues between the LSD1 inhibitor OG‐L002 group and the control group (*t*‐test). (C) Western blot analysis of CD74 expression in h22 cell line after LSD1 inhibitor OG‐L002 incubation for 48 h. (D) Representative IHC pictures of CD74 from harvested tumour tissues in Model 1. The violin map showed statistic results of number of CD74^+^ cells in tumour tissues between LSD1 inhibitor group and the control group (*n* = 6 for each group, **: *p* < .01, *t*‐test). (E, F) 5×10^6^ CD74 knock‐out (CD74^KO^) or wild type (WT) h22 cells in .1 mL of DMEM were injected subcutaneously in the flank of mice on Day 0 then treated the same as Model 1. Tumour growth volume was screened over time (*n* = 6 for each group, error bars represent SEM). Kaplan‒Meier analysis of tumour recurrence in different groups was also performed (log‐rank test).

To further address the mechanism underlying the induction of LSD1 deletion in preventing acquired resistance to anti‐PD1 immunotherapy, we conducted a thorough examination of the differentially expressed genes influenced by LSD1 inhibitor in both tumour cells and immune cells. As depicted in Figures 2G,H and 3A, LSD1 inhibitor primarily impacted cell cycle‐associated processes in liver cancer cells and exhibited a role in immune regulation and tumour antigen processing and presentation. Subsequently, we delved deeper into the mechanism of LSD1 inhibitor in remodelling relapse‐free niche in mouse HCC models mimicking different TME. A recent study^30^ has provided evidence suggesting that histone modifications induce resistance to tumour immunotherapy by affecting the antigen‐processing pathway. In our study, RNA‐seq data revealed a significant upregulation of 16 histocompatibility‐associated genes in the LSD1 inhibitor‐treated group compared to the NC in Model 1 (Figure 6A). Except for Mr1 and H2‐M3, 87.5% (14/16) genes mRNA were validated to be upregulated by *q*RT‐PCR, aligning with the results from mRNA‐seq (*p* < .01) (Figure 6B). Among these 14 validated genes, cd74 emerged as the top gene significantly influenced by LSD1 inhibitor treatment (Figure 6B). Western blotting further confirmed the increased expression of CD74 in h22 cells after LSD1 inhibitor treatment (Figure 6C). Using Model 1, we investigated the effect of LSD1 inhibitor on the expression of CD74, and IHC results demonstrated a significantly increased CD74 expression in LSD1 inhibitor‐treated xenografts (Figure 6D). Subsequently, we employed CRISPR/Cas‐9 to knock out CD74 in the h22 cell line (designated as CD74^KO^) (Figure S5), and then subcutaneously inoculated CD74^KO^ or (CD74^WT^) h22 cells as control into mice. After treatment with combined therapy of anti‐PD1 antibody and LSD1 inhibitor, complete tumour regression was observed in both CD74^KO^ and the control (CD74^WT^) group (Figure 6E). However, only CD74^KO^ group had tumour recurrence after withdrawal of the combined therapy of anti‐PD1 antibody and LSD1 inhibitor (Figure 6F).

CD74 plays a critical role in MHC class II antigen processing by stabilizing peptide‐free class II alpha/beta heterodimers in a complex. It also enhances the stimulation of T cell response through interaction with CD44.^31^ These data indicate that LSD1 inhibitor prevents from acquired resistance of PD1 blockage by activating MHC class I/II‐dependent antigen processing and presentation as well.

### 3.7 Advanced HCC patients expressing low LSD1, accompanied with high expression of CD74 and more effector CD8^+^ T cells show longer DoR to anti‐PD1 therapy

**FIGURE 7:**
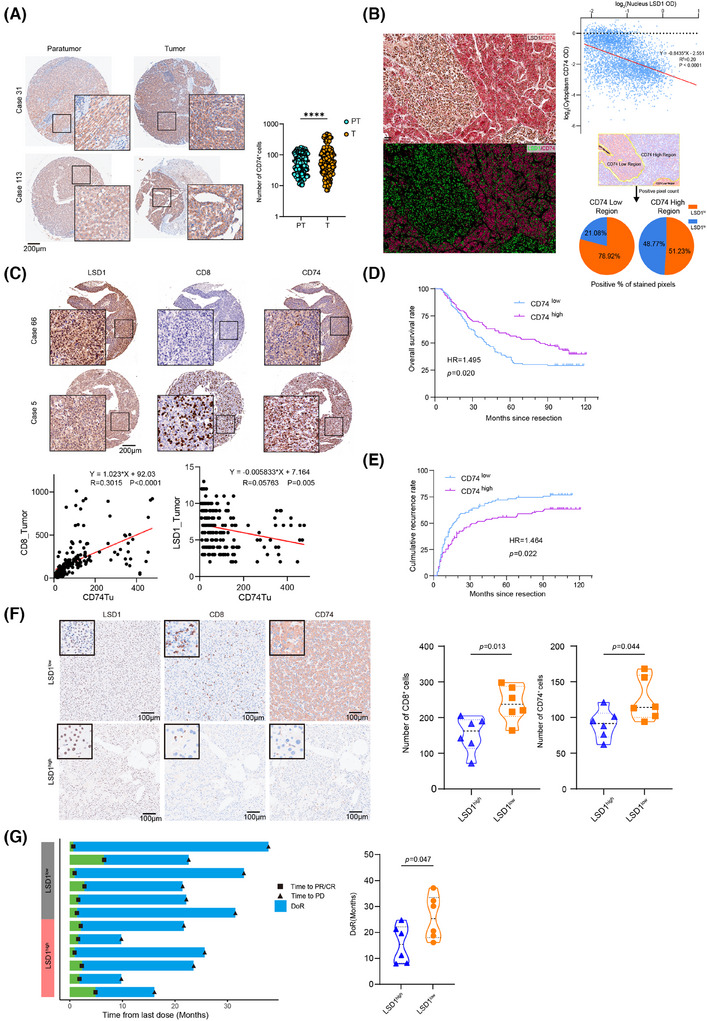
Advanced HCC patients with high CD74 showed durable tumour response to anti‐PD1 therapy. (A) Representative immunohistochemistry of CD74 in para‐tumour tissues (PT) and tumour tissues (T) in two HCC patients (Case 31 and Case 113). Statistic results of number of CD74^+^ cells between them also showed (*N* = 206, black line indicates Mean, ***: *p* < .001, *t*‐test was used). (B) Double‐staining immunohistochemistry indicated heterogeneity of CD74 (Red, nucleus) and LSD1 (Brown, cytoplasm) expression in a representative HCC patient (Left top). Pseudo‐colour staining clearly showed a negative correlation of staining intensity between LSD1 and CD74 (Left bottom). Statistic results showed a negative correlation between CD74 and LSD1 from 4881 cells (β = −.8435, *p *< .0001, *R* ^2 ^= .20, Right top). Statistic results show CD74^low^ region expressed LSD1^high^, and vice versa (Right bottom). (C) Two representative HCC patients (Case 5 and Case 66) showing different levels of LSD1, CD8 and CD74 expression. The correlations between CD74 expression and CD8 (β = 1.023, *p *< .0001, *R* ^2 ^= .30) or LSD1 (β = −.006, *p *= .005, *R* ^2 ^= .06) expression were calculated on protein level in HCC cohort (*N* = 206). (D, E) Kaplan−Meier estimate of overall survival and cumulative recurrence in the whole cohort (*N* = 206) with different levels of CD74 (log‐rank test). (F) Representative immunohistochemistry of LSD1, CD74 and CD8 in tumour tissues of 12 advanced HCC patients treated with anti‐PD1 therapy. The statistic results of number of CD8^+^ cells and CD74^+^ cells in tumour tissues between LSD1^high^ group and LSD1^low^ group (*n* = 6 for each group, presented as mean ± SEM, *t*‐test was used). (G) The time of achieving PR/CR, PD and DoR of 12 advanced HCC patients following their final administration of anti‐PD1 therapy (PR, partial response; CR, complete response; PD, progressive disease; DoR, duration of response). The patients are stratified into LSD1^high^ and LSD1^low^ groups based on the level of LSD1 expression demonstrated by immunohistochemistry (*n* = 6 for each group, *t*‐test was used).

We further examined the expression of CD74, LSD1 and effector CD8^+^ T cells in TMAs comprising 206 HCC patients. The results revealed predominant CD74 expression in the tumour cell, infiltrating lymphocytes and endothelial cells (Figure 7A). The expression of CD74 in para‐tumour tissue was lower than that in tumour tissue (63.16 ± 3.00 vs. 98.05 ± 7.57, *p* < .0001, Figure 7A). IHC demonstrated evident heterogeneity in CD74 expression among patients with HCC (Figure 7A). Intriguingly, even within the same patient, intra‐tumour heterogeneity of CD74 expression was notable as well (Figure 7B). Correlation analysis indicated that HCC patients with higher expression of CD74 in tumour tissue tended to exhibit lower expression of LSD1 and a higher number of CD8^+^ T cells (Figure 7C). Furthermore, the analysis of optical density of nucleus LSD1 (brown) and cytoplasmic CD74 (red) from all the 4881 cells in this representative field revealed a negative correlation between CD74 and LSD1 (β = −.8435, *p *< .0001, *R* ^2 ^= .20). Importantly, tumour regions with strong positive CD74 expression showed significantly lower LSD1 expression than those with weak CD74 expression (Figure 7B). In addition, HCC patients with higher CD74 expression in tumour tissue exhibited a better prognosis in terms of higher OS and lower cumulative recurrence rate (Figure 7D,E). We further conducted a retrospective analysis of 12 histologically confirmed patients with advanced HCC who received second‐line anti‐PD1 treatment after first‐line lenvatinib therapy. IHC results revealed that pre‐treatment, HCC patients with low LSD1 expression exhibited increased infiltration of effective CD8^+^ T cells and higher levels of tumour CD74 expression (Figure 7F). The median duration of response (mDoR) for these 12 advanced HCC patients was 20.1 months, with a range of 7.9−37.1 months. Among them, patients with high LSD1 expression in tumours had an mDoR of 15.4 months, whereas those with low LSD1 expression had an mDoR of 25.3 months. According to the statistic results, we found that patients with high LSD1 expression exerted significantly shorter DoR compared to the low LSD1 expression group (Figure 7G). These clinical findings indicate that low LSD1 expression levels lead to high expression of CD74, as well as more infiltration of effector CD8^+^ T cells, ultimately achieving a longer DoR in advanced HCC patients.

## 4 DISCUSSION

Consistent with previous studies,^11^, ^16^, ^19^ our study provided enough evidence to support the notion that LSD1 acts as a tumour‐promoting gene in HCCs. in vitro data revealed that LSD1 deletion inhibited tumour proliferation and induced the apoptosis of liver cancer cells. in vivo mouse model also showed that LSD1 deletion significantly impeded the tumour growth. Importantly, clinical data further demonstrated that HCC patients exhibiting low expression of LSD1 had a more favourable prognosis after radical resection, compared to those patients with high LSD1. Mechanistically, LSD1 deletion inhibited tumour growth through downregulation of cell cycle‐associated genes. Collectively, these data strongly suggest that LSD1 deletion unequivocally hinders the growth of liver cancer cells.

Clinical trials showed that only 16–18% of patients with advanced HCC earned survival benefits from PD1 blockade.^3^ Notably, updated data from KEYNOTE‐224 showed median DoR to anti‐PD1 therapy in advanced HCC was 21.0 months (range 3.1−39.5^+^ months),^32^ highlighting acquired resistance as a significant impediment to the long‐term survival of PD1 blockade‐sensitive patients. Recent studies have elucidated that histone demethylase LSD1 can suppress endogenous dsRNA and IFN responses, thereby rendering initially non‐responsive tumours sensitive to anti‐PD1 therapy.^11^ Intriguingly, our findings demonstrate that a combined therapy of LSD1 inhibitor and PD1 blockade has the potential to prevent acquired resistance to anti‐PD1 therapy in HCCs. On the one hand, as discussed in the aforementioned evidence, LSD1 deletion directly inhibited the growth of liver cancer cells. On the other hand, our data provided substantial evidence indicating that LSD1 deletion remodels the relapse‐free TME and serves as a preventive measure against acquired resistance to anti‐PD1 therapy in liver cancer.

First, in vitro findings revealed that LSD1 deletion led to an augmentation in the population of anti‐tumour immune cells, including CD8^+^ cells, CD4^+^ naive T cells and NK cells. Concurrently, it resulted in a reduction in the proportion of tumour‐promoting immune cells, specifically Treg cells, within PBMCs isolated from mice. Mechanistically, LSD1 deletion exerted its influence on the promoters of CD28 and GZMB, consequently enhancing the expression of these cytotoxic and co‐stimulatory molecules. The co‐stimulatory molecule CD28 plays a pivotal role in the proliferation, activation, cytokine production and development of CD8^+^ T cells. The downregulation of CD28 has been recognized as a hallmark of senescent T cells.^35^ Additionally, our data demonstrated that the LSD1 inhibitor elevated the concentration of GZMB in the supernatants of PBMCs. Furthermore, the proliferation of tumour cell was directly inhibited when tumour cells were co‐cultured with the supernatants of PBMCs pretreated with the LSD1 inhibitor.

Second, in vivo data further disclosed the multifaceted roles of the LSD1 inhibitor in enhancing the anti‐tumoral efficacy of the anti‐PD1 inhibitor. LSD1 inhibitor alone demonstrated definitely the capacity to impede tumour growth in tumour‐bearing mouse model. Critically, LSD1 inhibition activated several signalling pathways associated with inflammatory responses, T cell activation, positive immune response regulation, and antigen processing and presentation within xenografts. Furthermore, LSD1 inhibition induced a comprehensive reshaping of the TME in xenografts, characterized by the upregulation of PD‐L1 expression in tumour cells, increased infiltration of CD8^+^ CTLs and decreased infiltration of FoxP3^+^ Treg cells. Overexpression of PD‐L1 in tumour cells was considered as a predictor of the efficiency of anti‐PD1 therapy. in vivo, CD8^+^ CTLs, particularly GZMB^+^CD8^+^ T cells, serve as the primary anti‐tumour immune effectors by secreting crucial molecules such as GZMB and IFN‐γ. Treg cells, especially FoxP3^+^ cells, were considered as adverse predictors for several cancers, and the efficacy of PD1 blockade in intrahepatic tumours was recovered by targeting lactic acid metabolism of Treg cells.^36^ Taken together, these data suggested that LSD1 inhibition remodelled the anti‐tumoral immune microenvironment.

In prior investigations, Shi^13^ demonstrated that inhibiting LSD1 could transform a state of resistance to PD1 blockade into a sensitive condition in a mouse melanoma model. However, the impact of LSD1 inhibition on the anti‐tumoral effect of PD1 inhibitors depended on various factors. Therefore, in the present study, we constructed three mice models to emulate clinical scenarios and evaluated the responsiveness to anti‐PD1 within different TMEs. It was well acknowledged that the immune state emerged as another pivotal determinant for ICB responsiveness.^37^ In instances, tumour‐bearing mice being sensitive to anti‐PD1 antibody were administered with anti‐PD1 therapy and subsequently occurred to relapse, a compromised immune status was observed, characterized by diminished CD8^+^, CD4^+^ and GZMB^+^ levels. Conversely, naïve mice exhibited distinct anti‐tumoral immunity. As demonstrated in Model 3, the immune state of the tumour environment in mice before re‐transplant became exhausted, resulting in poor responses to anti‐PD1 antibody treatment. These findings underscore the influence of the immune state within the TME on the sensitivity of anti‐PD1 therapy. Consequently, reshaping the anti‐tumoral environment holds promise as an approach to extend the DoR in HCC patients resistant to anti‐PD1 therapy.

More interestingly, the present study showed LSD1 inhibitor demonstrated its potential in preventing from tumour relapse after the withdrawal of anti‐PD1 therapy in a tumour‐bearing mice model that exhibited sensitivity to anti‐PD1 therapy. Concurrently, clinical data revealed that advanced HCC patients with lower LSD1 levels experienced a prolonged DoR to anti‐PD1 therapy compared to patients expressing higher levels of LSD1. These effects are largely attributed to the role of LSD1 in reshaping the anti‐tumoral relapse's environment and enhancing antigen presentation related to CD74. Interestingly, a recent study found that in an IFN‐high immunophenotype of colorectal cancer that is sensitive to PD1 immunotherapy, CTLs can induce the upregulation of genes related to antigen processing and presentation, such as CD74, in adjacent tumour cells.^38^ Notably, although tumour‐bearing mice from CD74^KO^ HCC cells achieved complete tumour regression after the combined treatment of anti‐PD1 antibody and LSD1 inhibitor, tumour recurrence occurred after discontinuation of the combined therapy. Our clinical data further supported these observations, revealing that advanced HCC patients with lower LSD1 levels tended to exhibit higher CD74 expression and prolonged durable control following anti‐PD1 therapy. The elevation of CD74 expression in tumours induced by LSD1 deletion formed a multifaceted immune response environment, ultimately contributing to a sustained response to PD1 antibody therapy.

In conclusion, our study furnishes compelling evidence for the pivotal role of LSD1 in HCC development and elucidates the underlying mechanisms. Our findings offer insights into the mechanism by which tumour resistance to anti‐PD1 therapy was hindered by the use of LSD1 inhibitors. The combined application of LSD1 inhibitors and anti‐PD1 holds promise for mitigating acquired resistance to anti‐PD1 therapy in HCC patients. Clinical trials are imperative to unveil the role of combined therapy of LSD1 inhibitor and anti‐PD1 antibody in patients with HCC being sensitive to anti‐PD1 therapy.

## AUTHOR CONTRIBUTIONS

Concept and design: Jia Fan, Guo‐Ming Shi, Jia‐Cheng Lu and Zheng Tang; Data collection and analysis: Chen Liang, Peng‐Fei Zhang, Mu Ye, Lei Yu, Xiao‐Jun Guo, Xian‐Long Meng, Hai‐Ying Zeng, Shu‐Yang Hu, Dao‐Han Zhang, Qi‐Man Su, Ying‐Hao Shen, Jia‐Bin Cai, Shuang‐Qi Li, Zhen Chen, Ying‐Hong Shi, Ai‐Wu Ke, Yujiang G. Shi, Jian Zhou, Fei‐Zhen Wu, Xiao‐Yong Huang, Guo‐Ming Shi, Jia‐Cheng Lu, Jia Fan and Ai‐Wu Ke; Experiments: Chen Liang, Jia‐Cheng Lu, Peng‐Fei Zhang, Xiao‐Yong Huang, Xiao‐Jun Guo, Hai‐Ying Zeng, Qi‐Man Sun, Ying‐Hao Shen, Jia‐Bin Cai and Shuang‐Qi Li; Data analysis and visualization: Jia‐Cheng Lu and Fei‐Zhen Wu; Writing article: Chen Liang, Jia‐Cheng Lu, Guo‐Ming Shi and Peng‐Fei Zhang.

## CONFLICT OF INTEREST STATEMENT

The authors declare no competing interests.

## ETHICS APPROVAL AND CONSENT TO PARTICIPATE

Written informed consent was obtained from each patient. This study was approved by the Zhongshan Hospital Research Ethics Committee.

## Supporting information



Supporting Information

Supporting Information

Supporting Information

Supporting Information

Supporting Information

Supporting Information

Supporting Information

Supporting Information

Supporting Information

Supporting Information

Supporting Information

Supporting Information

Supporting Information

Supporting Information

Supporting Information

Supporting Information

Supporting Information

Supporting Information
