# Supplementary material for: Lysine‐specific demethylase 1 deletion reshapes tumour microenvironment to overcome acquired resistance to anti‐programmed death 1 therapy in liver cancer
Source: Clin Transl Med. 2025 May 12;15(5):e70335. doi: 10.1002/ctm2.70335 (PMC12069797; doi:10.1002/ctm2.70335)
Supplement: Supplementary file 1 — Supporting Information [file CTM2-15-e70335-s014.docx]

**Supplementary figure 1.**

(**A**) Relative mRNA level of LSD1 in h22 and hep1-6 cell line after different LSD1 shRNAs incubation. LSD-shRNA#1 was chosen to perform further experiment and renamed as ‘sh-LSD1’. (presented as mean ± SD, *: *p* < 0.05, **: *p* < 0.01, one-way ANOVA test, Tukey's multiple comparisons test for multiple comparison; WT: wild type).

(**B**) Immunofluorescence shows LSD1 expression in h22 and hep1-6 cell line after LSD1 shRNA incubation.
