# Supplementary figures and images for: Lysine‐specific demethylase 1 deletion reshapes tumour microenvironment to overcome acquired resistance to anti‐programmed death 1 therapy in liver cancer
Source: Clin Transl Med. 2025 May 12;15(5):e70335. doi: 10.1002/ctm2.70335 (PMC12069797; doi:10.1002/ctm2.70335)

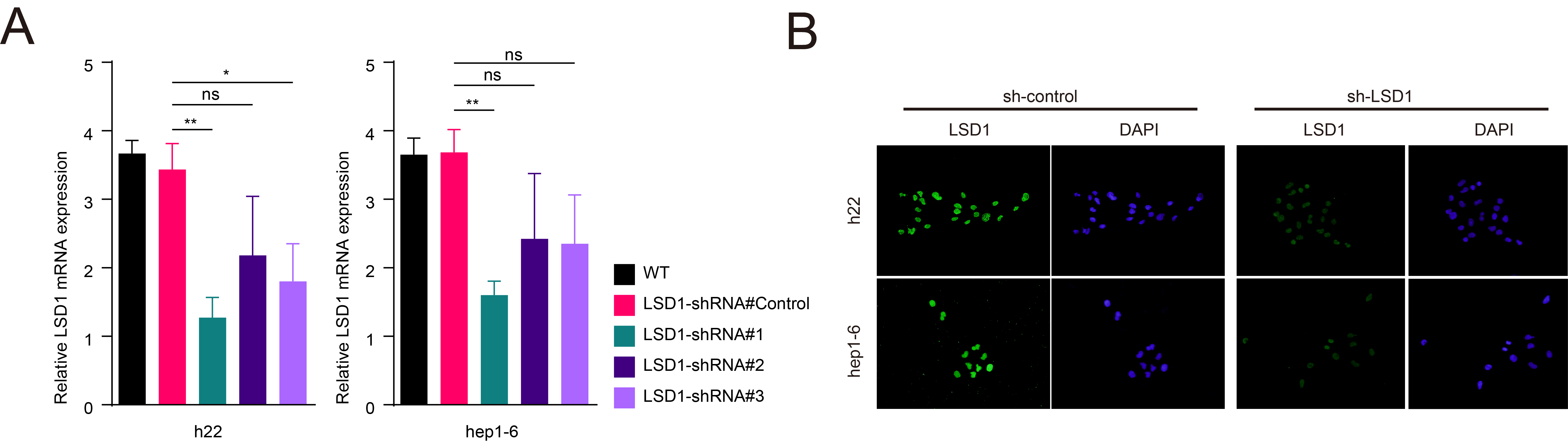

Supplement: Supplementary file 2 — Supporting Information [file CTM2-15-e70335-s008.png]

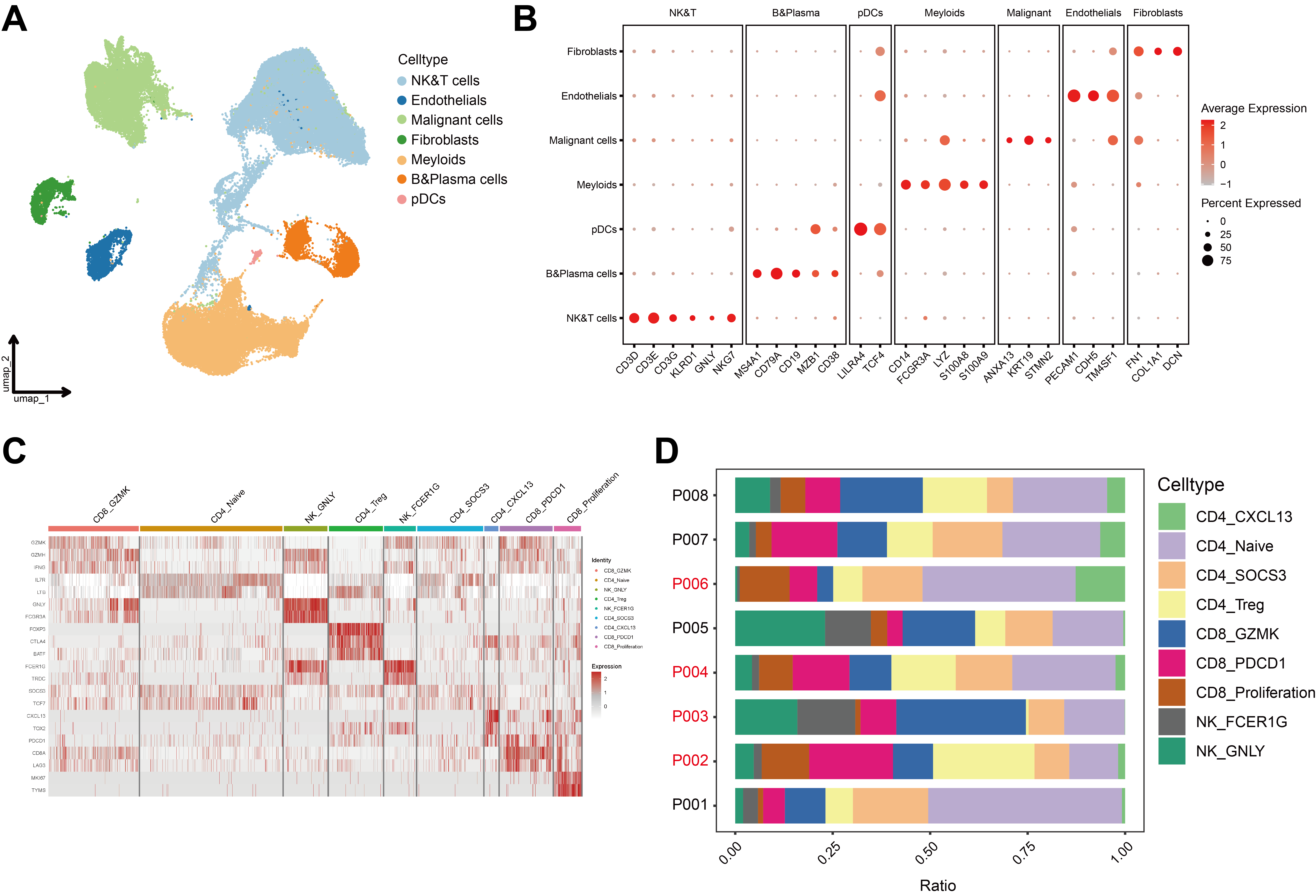

Supplement: Supplementary file 4 — Supporting Information [file CTM2-15-e70335-s001.png]

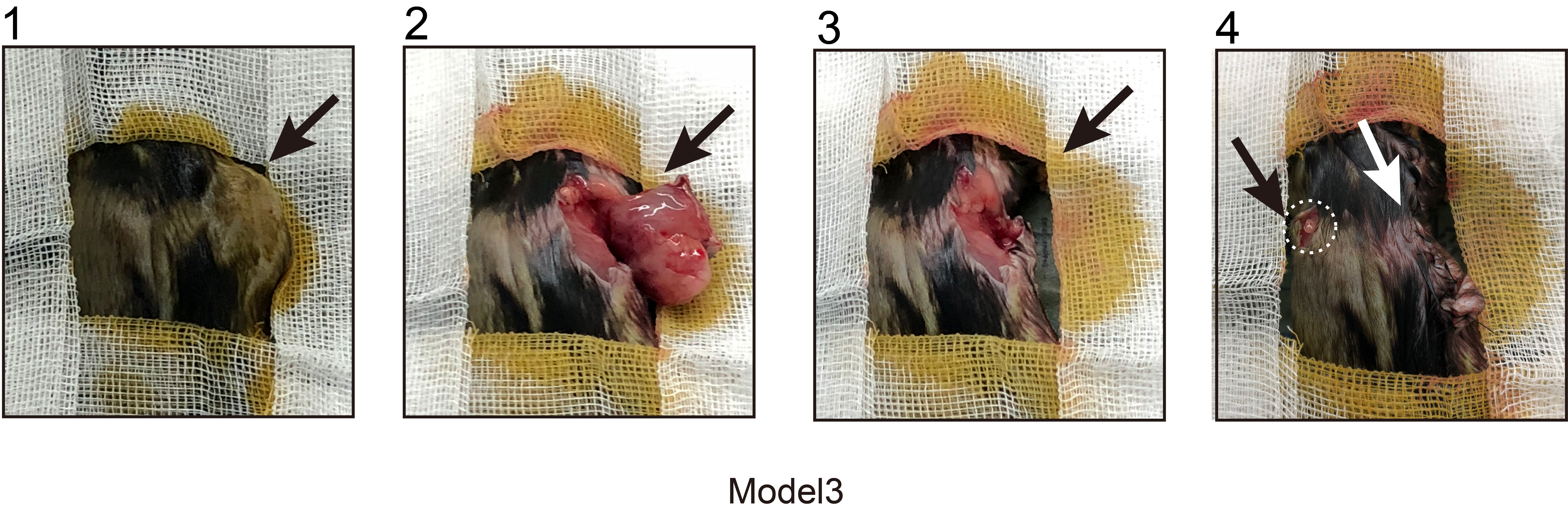

Supplement: Supplementary file 6 — Supporting Information [file CTM2-15-e70335-s005.png]

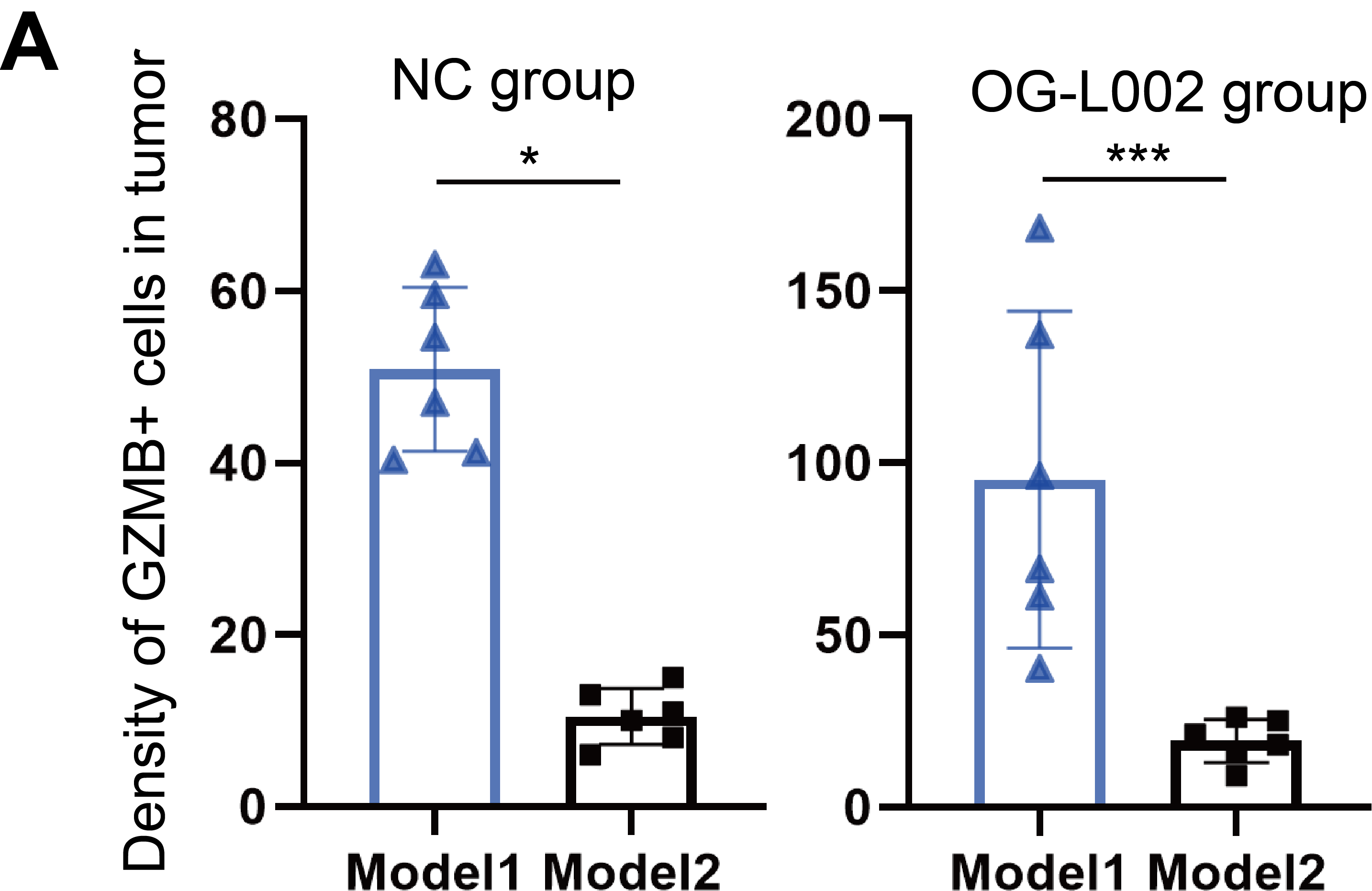

Supplement: Supplementary file 8 — Supporting Information [file CTM2-15-e70335-s007.png]

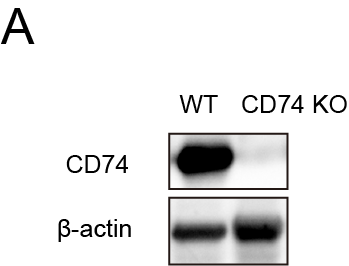

Supplement: Supplementary file 10 — Supporting Information [file CTM2-15-e70335-s003.png]
