# Supplementary material for: Lysine‐specific demethylase 1 deletion reshapes tumour microenvironment to overcome acquired resistance to anti‐programmed death 1 therapy in liver cancer
Source: Clin Transl Med. 2025 May 12;15(5):e70335. doi: 10.1002/ctm2.70335 (PMC12069797; doi:10.1002/ctm2.70335)
Supplement: Supplementary file 3 — Supporting Information [file CTM2-15-e70335-s004.docx]

**Supplementary Figure 2**

(**A**) Dimensionality reduction and clustering of scRNA-Seq data from 8 HCC patients, followed by manual annotation, identified 7 distinct cell clusters.

(**B**) Dot plot showing the marker genes for the 7 cell subpopulations.

(**C**) NK and T cells were further divided into 9 subclusters, with a heatmap displaying the marker genes for these subclusters.

(**D**) Proportions of the 9 subclusters among NK and T cells from the 8 patients. Patients highlighted in red represent the LSD1 high-expression group.
