# Supplementary material for: Lysine‐specific demethylase 1 deletion reshapes tumour microenvironment to overcome acquired resistance to anti‐programmed death 1 therapy in liver cancer
Source: Clin Transl Med. 2025 May 12;15(5):e70335. doi: 10.1002/ctm2.70335 (PMC12069797; doi:10.1002/ctm2.70335)
Supplement: Supplementary file 5 — Supporting Information [file CTM2-15-e70335-s013.docx]

**Supplementary figure 3.**

Pictures of crucial steps to construct Model 3.

Step 1: Recurrent tumors from PD1 antibody treatment group in Model 1;

Step 2-3: Completely remove the tumor;

Step 4: Re-transplanted 1×1 mm^3^ recurrent tumor into the contralateral subcutaneous of the same mouse (Black arrow: re-transplanted tumor; White arrow: original tumor position has been sutured).
