# Supplementary material for: Lysine‐specific demethylase 1 deletion reshapes tumour microenvironment to overcome acquired resistance to anti‐programmed death 1 therapy in liver cancer
Source: Clin Transl Med. 2025 May 12;15(5):e70335. doi: 10.1002/ctm2.70335 (PMC12069797; doi:10.1002/ctm2.70335)
Supplement: Supplementary file 7 — Supporting Information [file CTM2-15-e70335-s009.docx]

**Supplementary Figure 2**

Different density of GZMB+ cells in tumor showed by multiple-IHC staining between **Model 1** and **Model 2** from NC group or OG-L002 treatment group (presented as mean ± SD, *: p < 0.05, ***: p < 0.001, t-test).
