# Supplementary material for: Lysine‐specific demethylase 1 deletion reshapes tumour microenvironment to overcome acquired resistance to anti‐programmed death 1 therapy in liver cancer
Source: Clin Transl Med. 2025 May 12;15(5):e70335. doi: 10.1002/ctm2.70335 (PMC12069797; doi:10.1002/ctm2.70335)
Supplement: Supplementary file 9 — Supporting Information [file CTM2-15-e70335-s015.docx]

**Supplementary figure 3.**

CD74 knock-out (KO) in h22 via CRISPR-Cas9 was validated by western blotting (WT: Wild-type h22 cell line; CD74 KO: CD74 knock-out in h22).
