# Supplementary material for: Lysine‐specific demethylase 1 deletion reshapes tumour microenvironment to overcome acquired resistance to anti‐programmed death 1 therapy in liver cancer
Source: Clin Transl Med. 2025 May 12;15(5):e70335. doi: 10.1002/ctm2.70335 (PMC12069797; doi:10.1002/ctm2.70335)
Supplement: Supplementary file 11 — Supporting Information [file CTM2-15-e70335-s011.docx]

**Supplementary Materials and Methods**

**Isolation and culture of peripheral blood mononuclear cells (PBMCs) from mice**

PBMCs were obtained using Ficoll-Paque PLUS (GE Healthcare Biosciences) following the manufacturer's protocol with minor modifications. Briefly, 3mL blood of mice was diluted with 2-fold sterile PBS, and the 6mL suspension was poured over the 6 mL separation medium carefully. After 20 minutes of centrifugation at 1,500 rpm without brakes, the interphase was moved to a new tube, washed twice with PBS, and centrifuged for 4 min at 1,000 rpm. The resulting cell pellets were suspended in DMEM, adjusted to a concentration of 5 × 10^6^/mL. When performing LSD1 inhibitor associated experiment, 50 μM OG-L002 or corresponding DMSO (negative control) was added into the medium for 24h.

**RNA-seq data analysis**

mRNA sequencing were performed as our previous paper mentioned^25^. In brief, after total RNA was isolated using the TRIZOL reagent, RNA-seq library preparation was carried out according to manufacturer’s guidelines (Illumina).

For RNA-seq data analyses, first the FASTQ data of sequencing reads were trimmed using the program trim galore (v0.6.4) with parameters,’–paired –illumina’, to remove low quality reads and adapter reads. Then， the trimmed reads were mapped to the mouse genome refer-ence (UCSC mm9) using the software TopHat (v2.1.1) with default parameters. The program Cufflinks (v2.2.1) with default parameters was used to assign the mapped reads to mouse transcripts (UCSC mm9) for identification of the gene expression abundance, represented by FPKM (Fragments Per Kilobase of transcript per Mil-lion mapped reads). The FPKM values were normalized to TPM (Transcripts Per Kilobase Million) using the script, FPKM2TPM.R, to allow the comparison of gene expression between samples. The program cuffdiff with default parameters in the Cufflinks suite was used to calculate the fold-change and P-value of genes for comparison between our samples. Differentially expressed genes were investigated at the cut-off of fold-change >1.5. The R program from Bioconductor clusterProfiler (v3.14.0) or Metascape26 were used to perform GO term and KEGG pathway enrichment analysis for differential gene expression.

**scRNA-seq data analysis**

R 4.2.1 was used for the whole down-streaming scRNA-seq analysis. We assessed cell quality using the following criteria: (1) the number of total count per cell (library size) was below 50,000; (2) the number of detected genes was above 300 and below 7,500; (3) the percentage of mitochondrial genes was below 10; (4) the percentage of hemoglobin genes was below 0.1. After quality control, a total of 242,598 cells were retained for downstream analysis.

We merged the expression matrices of each sample and performed data integration using the har-mony package (version 0.1.1). Plotting of the integrated data revealed good mixing of immune and stromal cells between different samples, therefore, significant batch effects were excluded.

To extract useful information from single-cell data for model construction, each sample's single-cell data was performed dimensionality reduction and clustering analysis using the Seurat package (ver-sion 4.3.0). After scaling the sample data, we used the FindVariableFeatures function to select 2,000 highly variable genes, and then used the RunPCA function to calculate the top 50 principal components (PCs) of the data. We then selected the first 20 PCs and performed unsupervised clus-tering on the cells using the FindNeighbors and FindClusters functions (resolution=0.5). UMAP was used for dimensionality reduction of the sample data.
